# Peripheral blood CADM1 expression and multi-gene signatures in depressive disorders: a pilot case-control study from the United Arab Emirates

**DOI:** 10.3389/fpsyt.2026.1850124

**Published:** 2026-07-01

**Authors:** Rukhsana Nawaz, Eman Al Awadhi, Fadwa Al Mughairbi, Asma Bashir, Gabriel Andrade, Syed Ali Bokhari, Syed Fahad Javaid, Abdalla A. R. M. Hamid

**Affiliations:** 1 Department of Clinical Psychology, College of Medicine and Health Sciences, United Arab Emirates University, Al Ain, United Arab Emirates; 2 Department of Emergency Health, Fatima College of Health Sciences, Al Ain, United Arab Emirates; 3 Department of Psychology, College of Medicine, Ajman University, Ajman, United Arab Emirates; 4 Al Amal Psychiatric Hospital, Emirates Health Services, Dubai, United Arab Emirates; 5 Department of Psychiatry, College of Medicine and Health Sciences, United Arab Emirates University, Al Ain, United Arab Emirates

**Keywords:** CADM1, gene expression, major depressive disorder, peripheral blood biomarkers, United Arab Emirates

## Abstract

**Background:**

Depressive disorders are a leading cause of global disability, yet gene expression studies in Middle Eastern populations remain scarce. This study investigated peripheral blood expression of seven candidate genes, previously identified in animal models and early-onset depression, in adults with and without depressive disorders in United Arab Emirates (UAE).

**Methods:**

In a case-control design, 49 adults with depressive disorders (ICD-10 major depressive disorder, dysthymia, or adjustment disorder with depressed features) and 49 non-depressed controls were assessed with the Beck Depression Inventory-II and Patient Health Questionnaire-9. Peripheral blood expression of ADCY3, DGKA, FAM46A, CADM1, KIAA1539, MARCKS, and RAPH1 was measured by quantitative real-time PCR, normalised to GAPDH. Analyses included Mann-Whitney U tests, principal component analysis, fold-change estimation, pre-specified receiver operating characteristic analysis, and severity-restricted, age-balanced, and RNA-quality sensitivity analyses.

**Results:**

CADM1 showed modest upregulation in the depressive disorder group (1.30-fold; Mann-Whitney p = 0.049, uncorrected) that did not survive multiple testing correction (Benjamini-Hochberg p = 0.178). Principal component analysis revealed an unadjusted PC2 difference (p = 0.006), driven by CADM1 and RAPH1, that attenuated after age adjustment (MANCOVA p = 0.156). CADM1 correlated negatively with depression severity (BDI-II ρ = −0.280, p = 0.005), and the signal strengthened monotonically across severity strata (moderate-severe BDI-II ≥ 20, p = 0.029; severe BDI-II ≥ 29, p = 0.014), arguing against diagnostic heterogeneity as the primary driver. However, propensity score matching on age abolished the CADM1 effect (p = 0.965). Exploratory subgroup analyses suggested sex-specific patterns (CADM1 in males, p = 0.039; KIAA1539 in females, p = 0.010). The CADM1 area under the receiver operating characteristic curve was 0.616, indicating no diagnostic utility. *Post-hoc* power for individual genes was 5–19%.

**Conclusions:**

This pilot provides the first peripheral blood gene expression data for depression in a UAE population. Given the small sample, the 13-year mean age difference between groups, and unmeasured confounders, CADM1 is best framed as a hypothesis-generating candidate only; the signal was unstable after age balancing and the discrimination performance (AUC = 0.616) is below any clinically useful threshold. Validation in larger, strictly age-matched studies is required before any clinical interpretation.

## 1 Introduction

Depressive disorders represent one of the most prevalent mental health conditions globally, described as a disabling disorder associated with substantial emotional distress, severe interpersonal disruption, and increased risk for physical illness and death (1). Depressive disorders are characterised by symptoms that impair concentration, occupational performance, sleep, appetite, interpersonal relationships, and overall quality of life (2, 3). They impose significant costs upon individuals, healthcare systems, and society through elevated mortality, diminished work productivity, and reduced quality of life (4–6).

The biological basis of DD involves complex interactions among genetic, epigenetic, and environmental factors. Twin studies estimate the heritability of DD at 30–40%, suggesting a substantial genetic contribution (7–9). While genome-wide association studies have identified numerous risk loci (10, 11), translating these findings into mechanistic understanding requires examining gene expression in accessible tissues. Peripheral blood gene expression profiling has emerged as a promising approach, as blood transcriptomes partially reflect central nervous system changes and capture systemic immune-inflammatory alterations implicated in depression (12, 13).

Using a cross-species design, Pajer et al. (14) identified a panel of blood transcriptomic markers in an animal model of depression and validated several in adolescents with early-onset major depressive disorder (14). Redei et al. (13) subsequently confirmed differential expression of these markers in adult primary care patients undergoing cognitive behavioural therapy, and further developed the case for blood-based biomarker panels in depression (13, 15, 16). The seven genes carried forward into the present study were selected because each maps onto a biological pathway with prior evidence of relevance to depression, and together they constitute a validated panel rather than an *ad-hoc* selection.

CADM1 (cell adhesion molecule 1) encodes SynCAM1, a synaptic cell adhesion protein critical for synapse formation and maintenance (17, 18), and is implicated in social-emotional behavioural phenotypes in knockout mouse models (17) and in metabolic traits commonly comorbid with depression (19). ADCY3 (adenylyl cyclase 3) generates cyclic adenosine monophosphate downstream of G-protein-coupled receptors and has been mechanistically linked to depression and to the action of antidepressants targeting Gαs-coupled signalling (20). DGKA (diacylglycerol kinase alpha) regulates the diacylglycerol-to-phosphatidic acid switch in lipid signalling and was previously identified within blood transcriptomic biomarker panels in major depressive disorder (13). MARCKS (myristoylated alanine-rich C-kinase substrate) is a protein kinase C target involved in dendritic spine morphology and structural plasticity (21), processes consistently implicated in depression neurobiology. RAPH1 (Ras-associated and pleckstrin homology domains-containing protein 1) participates in actin-cytoskeleton remodelling, with relevance to neuronal morphology and synaptic remodelling. FAM46A (also termed TENT5A) is a non-canonical poly(A) polymerase implicated in immune-cell mRNA stability and inflammatory gene expression, with biological plausibility given the immune-inflammatory dimension of depression (12). KIAA1539 (also termed FAM214B) has been less mechanistically characterised but emerged from the same blood transcriptomic discovery work (13, 14). These seven genes together cover synaptic adhesion, second-messenger signalling, lipid signalling, cytoskeletal remodelling, structural plasticity, and immune-related mRNA regulation, mapping onto the principal biological domains in which peripheral expression changes have been reported in depression. We did not undertake *de novo* gene selection; rather, this panel was carried forward from established discovery work for validation in a previously unstudied population.

Despite growing global research on peripheral biomarkers in depression, the Middle Eastern and North African region remains substantially underrepresented. To our knowledge, this study is among the first peripheral blood gene-expression studies focused on major depressive disorder in a United Arab Emirates population, and one of the few from the region (21, 22). Cultural, environmental, and genetic differences may influence both disease presentation and molecular profiles, underscoring the need for region-specific data.

This pilot study aimed to: (1) compare peripheral blood expression of these seven candidate genes between adults with depressive disorders and non-depressed controls in the United Arab Emirates; (2) explore multi-gene expression patterns using principal component analysis; and (3) establish baseline molecular data to guide future, adequately powered studies in this population.

## 2 Materials and methods

### 2.1 Study design and participants

This cross-sectional, case-control study was conducted in Al Ain, Abu Dhabi, United Arab Emirates. The clinical group comprised 49 adults (≥ 18 years) with a current diagnosis of major depressive disorder, dysthymia, or adjustment disorder with depressed features, diagnosed by a psychiatrist using ICD-10 criteria, and recruited from the Behavioural Sciences Institute of Al Ain Hospital. These three categories were combined into a single depressive disorders group for two reasons. First, the candidate-gene panel under study was derived in cohorts that did not restrict to narrow major depressive disorder phenotypes; Pajer et al. (14) validated the panel in adolescents with early-onset major depressive disorder, and Redei et al. (13) extended validation to adult primary care patients with broadly defined depressive disorders undergoing cognitive behavioural therapy (13, 14, 21). A narrowly major-depressive-disorder-only design would therefore not match the population in which these markers were originally identified. Second, this pilot study was designed to evaluate the panel against a clinically meaningful contrast (any current depressive disorder versus no depressive disorder), which mirrors how a peripheral biomarker would be deployed in primary-care screening contexts where diagnostic precision is initially limited. Two pre-specified severity-stratified sensitivity analyses were performed to address the possibility of dilution by diagnostically milder cases: the clinical group was restricted first to participants with BDI-II ≥ 20 (a threshold for clinically significant moderate-to-severe depression) and then to participants with BDI-II ≥ 29 (the severe stratum, corresponding most closely to the symptom profile of a current major depressive episode).

Participants in the clinical group were excluded if they had (i) comorbid psychotic features, (ii) comorbid psychiatric disorders such as schizophrenia or personality disorders, or (iii) current substance use disorder. The non-depressed control group comprised 49 adults frequency-balanced on gender and nationality, recruited from a university community sample. Ethical approval was obtained from the Social Sciences Research Ethics Committee of the United Arab Emirates University (ERS_2018_5820) and the Al Ain Hospital Research Ethics Committee (AAHEC-02-19-007). Written informed consent was obtained from all participants.

### 2.2 Psychological assessments

Depressive symptoms were assessed using two validated instruments: the Beck Depression Inventory-II (BDI-II) (23), a 21-item self-report measure of depression severity (scores 0-63), and the Patient Health Questionnaire-9 (PHQ-9), a 9-item screening tool aligned with DSM criteria for major depression (scores 0-27) (24, 25). Both instruments have been validated in Arabic-speaking populations (26, 27).

### 2.3 Blood sample collection and RNA extraction

Peripheral venous blood (2.5 mL) was collected into PAXgene Blood RNA Tubes (PreAnalytiX, Hombrechtikon, Switzerland) and stored at −80 °C until processing. Total RNA was extracted using the PAXgene Blood RNA Kit (Qiagen, Hilden, Germany) according to the manufacturer’s protocol. RNA concentration and purity (260/280 and 260/230 absorbance ratios) were measured using a NanoDrop spectrophotometer (Thermo Fisher Scientific, Waltham, MA, USA). RNA integrity numbers from a Bioanalyzer or TapeStation platform were not available.

### 2.4 Quantitative real-time PCR

Complementary DNA was synthesised from extracted RNA using primers derived from TaqMan Array Tools (Thermo Fisher Scientific) (28). Quantitative real-time PCR was performed using an ABI 7500 system with SYBR Green chemistry. Each sample was assayed in triplicate for each gene, and the mean cycle threshold (CT) value across triplicates was used for all downstream analyses. Standard error of triplicates was recorded to assess intra-assay variability. Primer sequences for six of the seven target genes (ADCY3, DGKA, FAM46A, CADM1, MARCKS, RAPH1) are listed in **Supplementary Table 1**. Primer sequences for KIAA1539 and the endogenous reference gene GAPDH were not retrievable from laboratory records at the time of assay; this is a substantive reproducibility limitation, formally acknowledged in the Limitations section, that future replication work must address by re-deriving and reporting full primer sequences for all targets. All reactions were performed using validated commercial assays under standardised conditions. GAPDH was used as the sole reference gene; the implications for normalisation reliability are discussed in the Limitations section. Blood samples were collected at varying times of day depending on participant availability, and RNA extraction was performed across different processing batches; potential circadian and batch effects could therefore not be quantitatively assessed or corrected.

### 2.5 Statistical analysis

All statistical analyses were performed using Python 3.9 (scipy v1.11, statsmodels v0.14, scikit-learn v1.3) and verified independently. The significance level was set at α = 0.05 throughout.

#### 2.5.1 Quality control and sample exclusion

Prior to analysis, all samples were screened for data quality. RNA purity was assessed using NanoDrop spectrophotometry (260/280 and 260/230 absorbance ratios). All samples met the 260/280 ≥ 1.80 threshold. Six samples (5 depressive disorder, 1 non-depressed) had 260/230 ratios below 1.0; one of these (Patient 16) had already been excluded pre-analysis, indicating possible organic contamination; sensitivity analyses were conducted with and without these samples. One depressive-disorder sample (Patient 10) was excluded based on the following evidence: despite excellent RNA quality metrics (260/280 = 2.08, 260/230 = 2.07, concentration = 498.5 ng/μL, the highest in the dataset), this sample exhibited uniformly elevated CT values across all eight transcripts (z-scores 5.0–6.5 above the depressive-disorder group mean), including the reference gene GAPDH (CT = 26.5 versus group mean 17.5). Low triplicate variability (CT standard error 0.05–0.14 for six of seven target genes) confirmed these elevations were reproducible, ruling out random pipetting error. One target (ADCY3, CT = 38.8) yielded no triplicate standard error, indicating detection-limit amplification. The combination of excellent RNA purity with systematically elevated, reproducible CT values is consistent with reverse transcription failure or extreme cDNA dilution error. All analyses were conducted on the final sample of 48 depressive-disorder and 49 non-depressed participants.

#### 2.5.2 Primary group comparisons

Given violations of normality in the depressive-disorder group (Shapiro-Wilk p < 0.001 for all genes), Mann-Whitney U tests were used as the primary analysis, supplemented by Welch’s t-tests. Effect sizes were reported as Cohen’s d and Cliff’s delta (δ). Robust bootstrap regression (10,000 resamples) was additionally used to estimate group mean differences in Δ*C_T_* with non-parametric 95% confidence intervals (CIs), both unadjusted and adjusted for age. Benjamini-Hochberg (BH) false discovery rate correction was applied to all seven primary comparisons.

#### 2.5.3 Covariate adjustment

A significant age difference was observed between groups (depressive-disorder: 38.2 ± 10.8 years; non-depressed: 25.2 ± 6.0 years; p < 0.001). One-way ANCOVA models were fitted for each gene with group as the fixed factor and age as a covariate (**Supplementary Table 2**). Partial eta-squared (η²_p) was reported as the effect size measure.

#### 2.5.4 Fold-change estimation

Relative gene expression was calculated using the 2^-ΔΔ*c*^*_T_* method (29), where ΔΔ*C_T_* = mean Δ*C_T_* (depressive-disorder) − mean Δ*C_T_* (non-depressed). 95% CIs were derived from 10,000 bootstrap resamples.

#### 2.5.5 Principal component analysis

Prior to principal component analysis, a one-way multivariate analysis of variance (MANOVA) was conducted to test whether the seven-gene Δ*C_T_* profile differed between groups as a whole (Pillai’s trace). A MANCOVA was also run with age as covariate. Principal component analysis was then performed on standardised Δ*C_T_* values for all seven genes. Group differences in principal component scores were tested using both independent t-tests and Mann-Whitney U tests, and ANCOVA was used to assess whether principal component differences survived age adjustment.

#### 2.5.6 Receiver operating characteristic analysis

ROC analysis was pre-specified for the full panel of seven candidate genes as an exploratory diagnostic-discrimination check, not as a confirmatory diagnostic claim, and not contingent on which individual gene reached uncorrected significance. The rationale was that the panel was originally proposed as a candidate peripheral biomarker set (13, 14), and assessment of discrimination performance is a standard component of such evaluation, irrespective of whether any single gene reaches univariate significance. Reporting AUC values for the strongest candidates therefore quantifies how far this panel is from any clinically useful discrimination threshold (conventionally AUC ≥ 0.80), and serves to bound the practical translational ceiling of these markers in our sample. Receiver operating characteristic curves were constructed using Δ*C_T_* values as predictors of depressive-disorder status. AUC with 95% bootstrap CIs was calculated, and Youden’s index identified optimal classification thresholds.

#### 2.5.7 Logistic regression

Binary logistic regression was used to evaluate the discriminative capacity of the gene expression panel. Three models were compared: (A) seven gene Δ*C_T_* values only; (B) genes plus age and sex; and (C) age and sex only. Model C served as a benchmark to determine whether genes contributed classification information beyond demographics. All predictors were standardised. Ten-fold stratified cross-validation was used to estimate out-of-sample AUC, accuracy, sensitivity, and specificity. Stability was assessed across 100 random cross-validation seeds.

#### 2.5.8 Exploratory subgroup and sensitivity analyses

Sex-stratified and nationality-stratified Mann-Whitney U tests were conducted to explore effect modification. These were considered exploratory and not corrected for multiple comparisons. Five further sensitivity analyses were performed: (1) age-restricted analysis (ages 20–39, the overlap range); (2) full outlier removal (all samples with any gene Δ*C_T_* z-score > 3.0); (3) propensity score matching on age using nearest-neighbour matching with a caliper of 0.2 standard deviations of the propensity score (the most rigorous control for the age confound, at the cost of sample size); (4) quality-based sensitivity analysis excluding all samples with 260/230 ratios below 1.0; and (5) two pre-specified diagnostic-severity-restricted sensitivity analyses, in which the clinical group was restricted first to participants with BDI-II ≥ 20 (clinically significant moderate-to-severe depression) and then to BDI-II ≥ 29 (the severe stratum, corresponding most closely to the symptom profile of a current major depressive episode), thereby excluding the milder clinical sub-strata most likely to capture dysthymia or adjustment disorder with depressed features. Age-adjusted analysis of covariance was additionally performed within the severe stratum to test whether the severity-based strengthening was independent of age. These analyses were added to address the concern that the broad depressive-disorder grouping might dilute a major-depressive-disorder-specific signal.

#### 2.5.9 RNA quality and triplicate variability assessment

RNA concentration and purity (260/280 and 260/230 absorbance ratios) were measured by NanoDrop spectrophotometry for all samples. Group differences were tested using Mann-Whitney U. Samples failing the 260/230 < 1.0 threshold were flagged for sensitivity analysis. Intra-assay coefficients of variation were calculated from triplicate CT standard errors as coefficient of variation (%) = (CT standard error × √3/CT mean) × 100.

#### 2.5.10 Gene expression and depression severity correlations

Spearman rank correlations were computed between each gene’s Δ*C_T_* and individual BDI-II and PHQ-9 scores. Two approaches were used: (1) within the DD group only (N = 48), to assess dose-response within clinically depressed individuals; and (2) across the full sample (N = 97), to assess the dimensional relationship between gene expression and depression severity regardless of diagnostic category. The full-sample analysis is the more powerful approach, as it captures the full range of depression scores. Benjamini-Hochberg correction was applied across all 14 tests (7 genes × 2 measures).

#### 2.5.11 *Post-hoc* power

*Post-hoc* power was calculated for each gene based on observed effect sizes (N = 48 vs. 49, α = 0.05). Required sample sizes for 80% power were estimated.

## 3 Results

### 3.1 Participant characteristics

**Figure 1:**
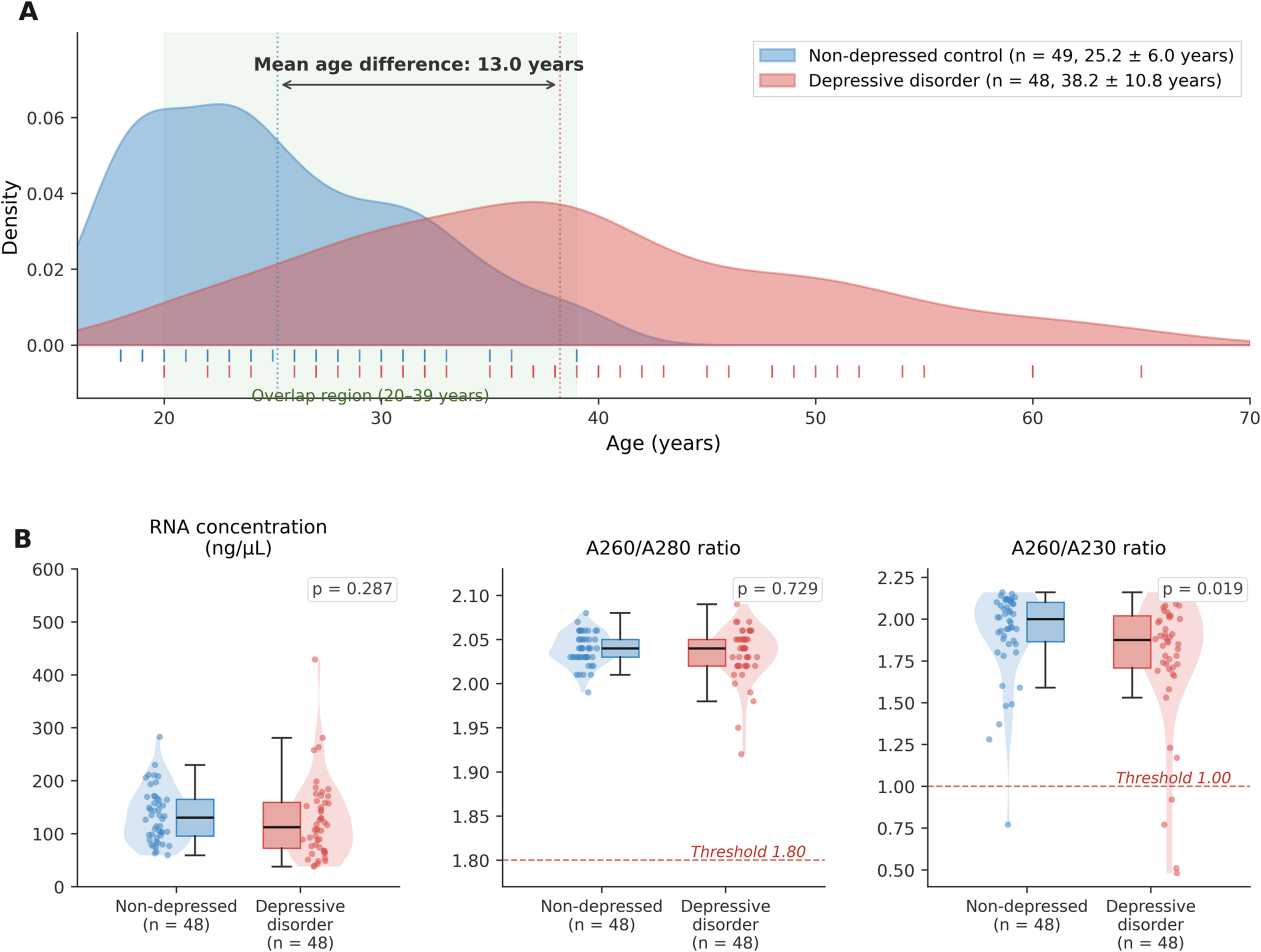
Sample characteristics and quality overview. **(A)** Age distribution of the depressive-disorder and non-depressed control groups, shown as kernel density estimates with individual participant ages indicated by tick marks (rugplot) below each density. Dotted vertical lines mark group means (25.2 years for non-depressed; 38.2 years for depressive-disorder), and the annotated arrow indicates the 13.0-year mean age difference. Shaded green region indicates the age-overlap range (20–39 years). **(B)** RNA quality metrics by group: RNA concentration (ng/μL), 260/280 absorbance ratio, and 260/230 absorbance ratio. Each panel shows violin plots (representing the underlying distribution), box plots (median, interquartile range, and whiskers), and individual data points with horizontal jitter. Red dashed lines indicate accepted quality thresholds (260/280 ≥ 1.80; 260/230 ≥ 1.00). The depressive-disorder group had significantly lower 260/230 ratios (p = 0.019), although all samples met the 260/280 threshold. p-values from Mann-Whitney U tests.

A total of 100 participants were enrolled: 50 allocated to the clinical group and 50 to the control group. Two participants (Patient 16 and Control 81) were excluded prior to data analysis (no questionnaire or molecular data available), yielding 49 per group. Depression severity scores (BDI-II, PHQ-9) were computed for this full 49-per-group sample. NanoDrop spectrophotometry data were available for 49 of 49 clinical and 48 of 49 control participants (Control 51 lacked NanoDrop data); RNA-quality analyses therefore used 49 depressive-disorder and 48 non-depressed participants. Gene-expression analyses used 48 depressive-disorder and 49 non-depressed participants after exclusion of Patient 10 for reverse transcription failure (Control 51 had usable CT data despite lacking NanoDrop data). The quality-based sensitivity analysis further excluded samples with 260/230 < 1.0, yielding 44 depressive-disorder and 48 non-depressed participants. Demographic characteristics are shown in **Table 1**. Groups were comparable in gender (χ² = 0.010, p = 0.922) and nationality (p = 0.636) but differed significantly in age (depressive-disorder: 38.2 ± 10.8; non-depressed: 25.2 ± 6.0; p < 0.001; **Figure 1A**). Depression severity was significantly higher in the depressive-disorder group: BDI-II (32.49 ± 10.74 versus 5.45 ± 3.51; p < 0.001) and PHQ-9 (14.80 ± 4.38 versus 2.45 ± 1.62; p < 0.001; **Table 2**; **Supplementary Table 3**).

**Table 1:** Demographic characteristics of the depressive-disorder (n = 48) and nondepressed groups (n = 49).

Characteristic	DD (n = 48)	ND (n = 49)	P-value
Age (years) mean ± SD	38.2 ± 10.8	25.2 ± 6.0	**<0.001**
Range	20–65	18–39	
Gender, n (%)			0.922
Male	24 (50.0)	26 (53.1)	
Female	24 (50.0)	23 (46.9)	
Nationality, n (%)			0.636
Emirati	22 (45.8)	20 (40.8)	
Expatriate	26 (54.2)	29 (59.2)	

Age compared by independent-samples t-test; gender and nationality compared by chi-square test. Sample comprises all enrolled participants with complete demographic data (Patient 16 and Control 81 were excluded pre-analysis owing to missing questionnaire and molecular data). SD, standard deviation. Bold indicates statistical significance (p < 0.05).

**Table 2:** Depression severity scores by group (n = 49 per group).

Measure	Group	N	Mean	SD	t	df	p
BDI-II	DD	49	32.49	10.74	16.84	96	≤0.001
	ND	49	5.45	3.51			
PHQ-9	DD	49	14.80	4.38	18.61	96	≤0.001
	ND	49	2.45	1.62			

BDI-II, Beck Depression Inventory-II (score range 0–63); PHQ-9, Patient Health Questionnaire-9 (score range 0–27). Depression severity was computed from the full pre-quality-control sample, since clinical scoring is independent of subsequent gene-expression quality control. Patient 10 was retained in this table and excluded only from gene-expression analyses on technical sample-failure grounds (see Methods). df, degrees of freedom; SD, standard deviation.

### 3.2 Sample quality control

One depressive-disorder participant (Patient 10) was excluded based on quality control criteria. NanoDrop data were available for 48 of 49 controls and all 49 clinical participants in the gene expression dataset; analyses used 49 non-depressed and 48 depressive-disorder participants after exclusion of Patient 10. RNA quality assessment revealed no significant differences between groups in concentration (control: 133.6 ± 50.4 ng/μL; depressive-disorder: 133.8 ± 90.5 ng/μL; p = 0.287) or 260/280 ratio (control: 2.04 ± 0.02; depressive-disorder: 2.03 ± 0.03; p = 0.729). The depressive-disorder group had significantly lower 260/230 ratios (control: 1.92 ± 0.27; depressive-disorder: 1.77 ± 0.40; p = 0.019), with five samples in the analytic dataset (four depressive-disorder, one non-depressed; a sixth low-260/230 sample, Patient 16, had already been excluded pre-analysis) falling below the 1.0 threshold (**Figure 1B**, **Table 3**, **Supplementary Table 4**). All samples passed the 260/280 ≥ 1.80 threshold. Intra-assay triplicate variability was acceptable across all genes (mean coefficient of variation: 0.86–1.41%; **Supplementary Table 5** and S6), confirming technical reproducibility. Five samples had missing CT standard error values for one gene each, indicating a single replicate dropout; these did not affect downstream analyses. GAPDH inter-sample coefficient of variation was 8.0% (after Patient 10 exclusion), above the recommended 5% but within the range reported for blood-based studies.

**Table 3:** RNA quality metrics by group (49 depressive-disorder, 48 non-depressed).

Metric	Control (n=48)	DD (n=49)	MW p	Threshold
Concentration (ng/μL)	133.6 ± 50.4	133.8 ± 90.5	0.287	—
260/280 ratio	2.04 ± 0.02	2.03 ± 0.03	0.729	≥1.80
260/230 ratio	1.92 ± 0.27	1.77 ± 0.40	0.019*	≥1.00

Values are mean ± standard deviation. Sample comprises participants with available NanoDrop spectrophotometry data: 49 of 49 depressive-disorder participants and 48 of 49 non-depressed controls (Control 51 lacked NanoDrop data). Patient 10 is retained in this table because RNA quality metrics were within range; this participant was excluded only from downstream gene-expression analyses owing to reverse-transcription failure across all eight transcripts (see Methods). Threshold values follow standard laboratory practice (260/280 ≥ 1.80; 260/230 ≥ 1.00). **Bold** indicates statistical significance (p < 0.05).

### 3.3 Gene expression: group comparisons

**Figure 2:**
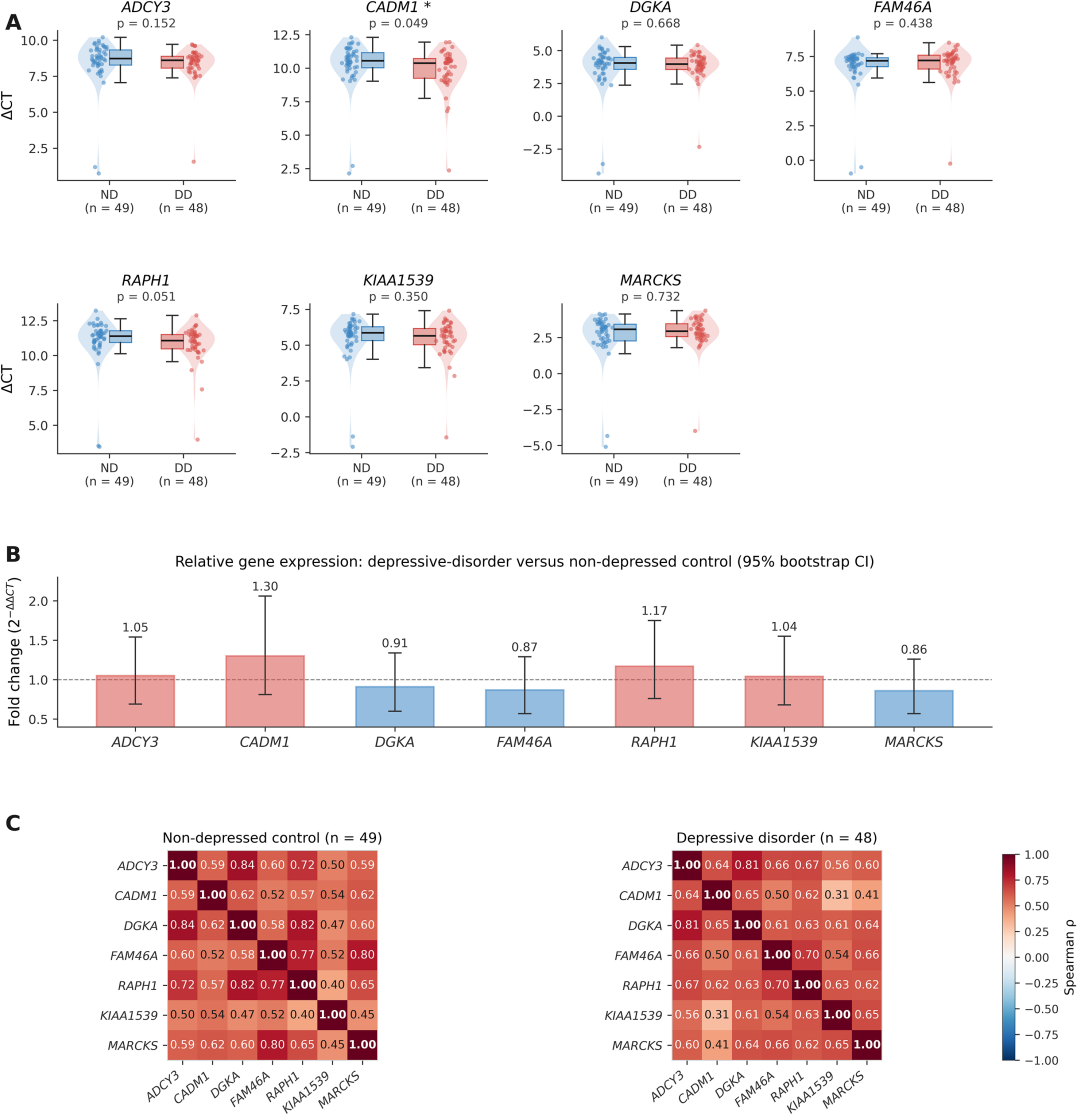
Gene expression patterns across seven candidate genes. **(A)** Δ*C_T_* distributions for each of the seven candidate genes by group, shown as violin plots, box plots (median, interquartile range, and whiskers), and individual data points with horizontal jitter. Sample sizes: non-depressed control (ND) n = 49, depressive disorder (DD) n = 48. Lower Δ*C_T_* values indicate higher gene expression. p-values from Mann-Whitney U tests are reported as displayed; an asterisk indicates uncorrected significance at the conventional p < 0.05 threshold. No comparison survived Benjamini-Hochberg false discovery rate correction across the seven primary tests. **(B)** Relative gene expression (Fold change was calculated as 2^-ΔΔ*C*^_*T*_ ).{-ΔΔ*C_T_*}−ΔΔ*C_T_*) in the depressive-disorder versus non-depressed control group, with point estimates labelled above each bar and 95% confidence intervals derived from 10,000 bootstrap resamples. The dashed horizontal line indicates no change (fold = 1.0); red fill indicates fold change > 1 (higher expression in the depressive-disorder group), blue fill indicates fold change < 1 (lower expression). All 95% confidence intervals cross 1.0, consistent with no significant differential expression after rigorous estimation. **(C)** Gene co-expression heatmaps showing Spearman rank correlation (ρ) matrices among the seven candidate genes within the non-depressed control group (left, n = 49) and within the depressive-disorder group (right, n = 48). Cell values are Spearman ρ; the diagonal (always 1.00) is bolded for orientation.

No significant differences in Δ*C_T_* values were detected after Benjamini-Hochberg correction (**Table 4**). CADM1 showed the strongest signal (Mann-Whitney p = 0.049; Cohen’s d = −0.23; Cliff’s δ = 0.23; **Table 4**), though this did not survive correction (adjusted p = 0.178). Robust bootstrap regression confirmed a non-significant group difference for CADM1 (β = −0.384, 95% bootstrap CI: −1.045 to +0.294, p = 0.254; **Supplementary Table 7**). After age adjustment, bootstrap estimates were further attenuated (CADM1 β = −0.385, 95% CI: −1.059 to +0.211). Both the parametric ANCOVA (p = 0.378) and the non-parametric age-adjusted bootstrap regression (p = 0.202; **Table 1**) were concordantly null for CADM1 after age adjustment. No other gene approached significance by any method (**Figure 2A**).

**Table 4:** Gene expression (Δ*C_T_*) comparisons between the depressive-disorder and non-depressed groups (48 depressive-disorder, 49 non-depressed).

Gene	ND Mean ± SD	DD Mean ± SD	U	p (U)	Welch t	p (t)	d	δ	p (BH)
ADCY3	8.47 ± 1.71	8.41 ± 1.17	1375	0.152	0.216	0.829	−0.04	−0.17	0.355
CADM1	10.28 ± 1.82	9.90 ± 1.57	1449	0.049*	1.112	0.269	−0.23	−0.23	0.178
DGKA	3.74 ± 1.79	3.87 ± 1.10	1236	0.668	-0.437	0.664	+0.09	−0.05	0.732
FAM46A	6.78 ± 1.66	6.98 ± 1.26	1068	0.438	-0.678	0.500	+0.14	**+0.09**	0.613
RAPH1	11.08 ± 1.73	10.86 ± 1.35	1447	0.051	0.706	0.482	−0.14	−0.23	0.178
KIAA1539	5.50 ± 1.66	5.45 ± 1.35	1306	0.350	0.175	0.862	−0.04	−0.11	0.613
MARCKS	2.67 ± 1.68	2.89 ± 1.19	1128	0.732	-0.744	0.459	+0.15	+0.04	0.732

DD, depressive-disorder group; ND, non-depressed control group; Δ*C_T_*, delta cycle threshold; CI, confidence interval; BH, Benjamini–Hochberg; FDR, false discovery rate. p-values are from Mann–Whitney U tests unless otherwise specified. Cohen’s d was calculated as DD minus ND. Cliff’s δ; was calculated with DD as the first group and ND as the second group. Negative values indicate lower Δ*C_T_* values in the DD group, corresponding to higher relative expression. BH-adjusted p-values were calculated across the seven primary gene comparisons. Bold indicates statistical significance at p < 0.05.

### 3.4 Fold-change analysis

CADM1 showed the largest fold change (1.30-fold upregulation in the depressive-disorder group; 95% CI: 0.81-2.06), followed by RAPH1 (1.17-fold; 95% CI: 0.76-1.75). All 95% CIs crossed 1.0 (**Tables 4**, **5**; **Figure 2B**).

**Table 5:** Relative gene expression (fold change) in the depressive-disorder versus non-depressed groups (48 depressive-disorder, 49 non-depressed).

Gene	Δ*C_T_* (ND)	Δ*C_T_* (DD)	ΔΔ*C_T_*	Fold	95% CI	Direction
ADCY3	8.47	8.41	-0.06	1.05	0.69–1.54	≈Unchanged
CADM1	10.28	9.90	-0.38	1.30	0.81–2.06	Upregulated
DGKA	3.74	3.87	0.13	0.91	0.60–1.34	Downregulated
FAM46A	6.78	6.98	0.20	0.87	0.57–1.29	Downregulated
RAPH1	11.08	10.86	-0.22	1.17	0.76–1.75	Upregulated
KIAA1539	5.50	5.45	-0.05	1.04	0.68–1.55	≈Unchanged
MARCKS	2.67	2.89	0.22	0.86	0.57–1.26	Downregulated

Column headers ND, non-depressed control; DD, depressive-disorder group. Sample excludes one depressive-disorder participant (Patient 10) on the basis of technical sample failure (see Methods). Fold change, 2^−ΔΔ*C*_*T*_^, where ΔΔ*C_T_*, (mean Δ*C_T_* depressive-disorder) minus (mean Δ*C_T_* non-depressed control). CI, confidence interval. 95% CIs were derived from 10,000 bootstrap resamples. All fold-change 95% CIs cross 1.0, consistent with no significant differential expression after rigorous estimation.

### 3.5 Principal component analysis

MANOVA indicated that the seven-gene Δ*C_T_* profile differed significantly between groups (Pillai’s trace = 0.199, F(7, 89) = 3.16, p = 0.005). However, when age was included as a covariate (MANCOVA), the group effect was no longer significant (Pillai’s trace = 0.111, F(7, 88) = 1.57, p = 0.156), consistent with the pattern observed for individual genes.

PC1 captured 90.3% of variance with approximately equal gene loadings, likely reflecting shared technical variation (RNA input, reverse transcription efficiency) rather than biology (**Figure 2C**). PC1 did not differ between groups (p = 0.97).

PC2 captured 3.5% of variance and showed a significant group difference (U = 1555, p = 0.006; t = 2.94, p = 0.004), loaded primarily on CADM1 (0.75) and RAPH1 (0.33) (**Supplementary Table 8**). However, ANCOVA controlling for age attenuated this effect (p = 0.13), indicating partial confounding.

### 3.6 Exploratory subgroup analyses

In males (n = 26 non-depressed, 24 depressive-disorder), CADM1 was significant (p = 0.039; d = −0.40); in females (n = 23 non-depressed, 24 depressive-disorder), CADM1 was non-significant (p = 0.558), but KIAA1539 reached exploratory, uncorrected significance (p = 0.010; d = −0.34; **Table 6**; **Supplementary Table 9**). Among Emiratis (n = 20 non-depressed, 22 depressive-disorder), CADM1 was significant (p = 0.029; d = −0.17); among expatriates (n = 29 non-depressed, 26 depressive-disorder), it was not (p = 0.376; d = −0.26).

**Table 6:** Sensitivity and exploratory subgroup analyses for CADM1.

Analysis	N (ND)	N (DD)	p (U)	d	Finding
Full sample (P10 excluded)	49	48	0.049	−0.23	Borderline significant
Age-restricted (20–39)	38	29	0.431	−0.23	NS (underpowered)
Full outlier removal	47	47	0.025*	−0.57	Effect strengthens
Males only	26	24	0.039*	−0.40	Significant
Females only	23	24	**0.558**	−0.02	No effect
Emiratis only	20	22	0.029*	−0.17	Significant
Expatriates only	29	26	**0.376**	−0.26	NS
QC-filtered (260/230≥1.0)	48	44	0.031*	−0.26	Signal strengthens
Propensity-matched (age)	23	23	0.965	−0.03	Signal lost (age balanced)

Column headers: ND, non-depressed control; DD, depressive-disorder group. All analyses are based on the gene-expression analytic sample after exclusion of one depressive-disorder participant, Patient 10, on the basis of technical sample failure. n values shown per row reflect the specific subgroup or sensitivity restriction applied. All p-values are from Mann–Whitney U tests and are uncorrected. d = Cohen’s d, calculated as depressive-disorder minus non-depressed; negative d indicates lower Δ*C_T_*, corresponding to higher relative expression in the depressive-disorder group. All subgroup analyses are exploratory and uncorrected for multiple comparisons. See **Supplementary Table 14** for all seven genes across the same subgroups. Bold indicates statistical significance, p < 0.05.

### 3.7 ROC analysis

**Figure 3:**
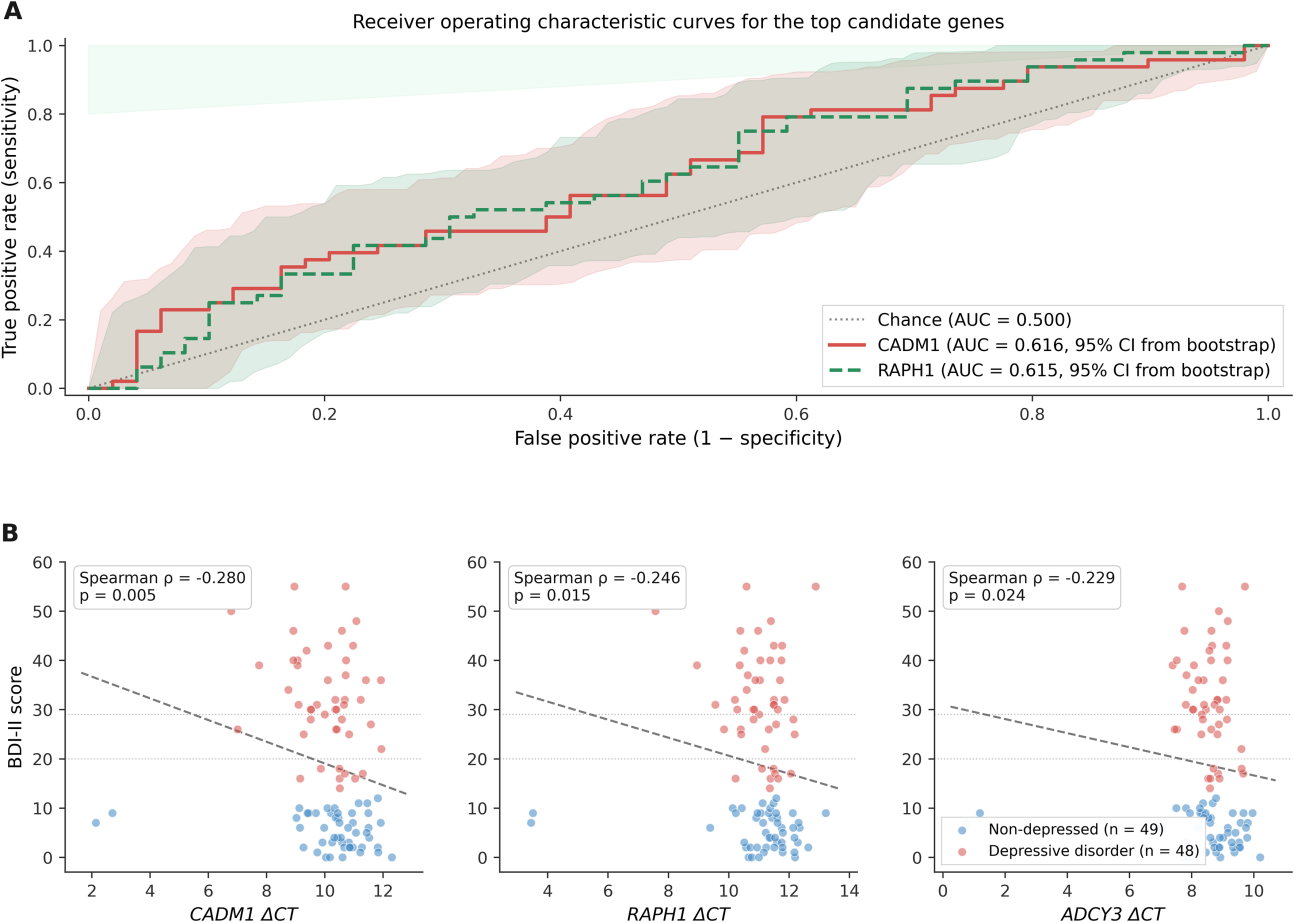
Diagnostic discrimination and dimensional associations with depression severity. **(A)** Receiver operating characteristic curves for the top two candidate genes (CADM1 and RAPH1) as predictors of depressive-disorder status using Δ*C_T_* values. Shaded bands around each curve represent 95% confidence intervals derived from 200 bootstrap resamples. The dotted diagonal line indicates chance performance (AUC = 0.500). The shaded green region at the top of the plot indicates the acceptable-discrimination zone (AUC ≥ 0.80); both candidate-gene curves fall well below this threshold, conventionally regarded as indicative of acceptable diagnostic discrimination, indicating no clinically useful discrimination performance in the present sample. **(B)** Scatter plots of gene expression (Δ*C_T_*) versus BDI-II score for the three genes with significant uncorrected Spearman correlations (CADM1, RAPH1, ADCY3). Blue circles indicate non-depressed control participants (n = 49); red circles indicate depressive-disorder participants (n = 48). The dashed grey line represents the least-squares regression line across the full sample (n = 97); inset Spearman rank correlation coefficients (ρ) and p-values are computed across the same full sample. The dotted horizontal lines mark the two BDI-II severity thresholds used in the pre-specified sensitivity analyses (BDI-II = 20 for moderate-to-severe; BDI-II = 29 for severe).

CADM1 yielded an AUC of 0.616 (95% CI: 0.500-0.725; sensitivity 79.2%, specificity 42.9%). RAPH1 was similar (AUC = 0.615; **Figure 3A**). Both values fall well below the AUC ≥ 0.80 threshold conventionally regarded as indicative of acceptable diagnostic performance, and the lower bound of the CADM1 CI touches the chance-classification line (AUC = 0.500). These curves therefore quantify a near-absence of diagnostic utility in this sample and should not be interpreted as supporting clinical translation; they are presented because evaluation of discrimination performance was pre-specified for the candidate panel.

### 3.8 Logistic regression

Logistic regression with 10-fold cross-validation revealed that the seven-gene panel alone (Model A) achieved a mean AUC of 0.717 ± 0.016, accuracy of 67.6%, sensitivity of 62.5%, and specificity of 73.5% (**Supplementary Table 10**). Adding age and sex (Model B) improved performance substantially (AUC = 0.854 ± 0.007, accuracy 78.6%). However, age and sex alone without gene data (Model C) achieved nearly identical performance (AUC = 0.848 ± 0.003, accuracy 76.9%). The marginal improvement from adding genes (ΔAUC = 0.006) confirms that the gene panel has no practical discriminative utility beyond demographic variables in this sample and indicates that age, not gene expression, drives classification. Within the genes-only model, RAPH1 (β = −1.271) and FAM46A (β = +1.161) had the largest standardised coefficients (**Supplementary Table 11**).

### 3.9 Gene expression and depression severity

Across the full sample (n = 97), CADM1 Δ*C_T_* showed the strongest negative correlation with BDI-II scores (Spearman ρ = −0.280, p = 0.005) and PHQ-9 scores (ρ = −0.243, p = 0.017), indicating that higher CADM1 expression was associated with greater depression severity (**Figure 3B**; **Supplementary Table 12**). RAPH1 also correlated with BDI-II (ρ = −0.246, p = 0.015) and showed a trend with PHQ-9 (ρ = −0.198, p = 0.052). ADCY3 showed a similar pattern (BDI-II ρ = −0.229, p = 0.024). After Benjamini-Hochberg correction across 14 tests, CADM1 and RAPH1 remained at trend level (adjusted p = 0.076–0.083). Within the depressive-disorder group alone (n = 48), correlations were weaker (CADM1 BDI-II ρ = −0.248, p = 0.090; FAM46A BDI-II ρ = −0.247, p = 0.091), as expected given the restricted range of depression scores (**Supplementary Table 13**).

### 3.10 Sensitivity analyses

In the age-restricted subset (20–39 years; 38 non-depressed, 29 depressive-disorder), no gene reached significance (all p > 0.17), though power was severely reduced. In the full outlier-removal analysis (3 outlier samples excluded beyond Patient 10; 47 non-depressed, 47 depressive-disorder), CADM1 strengthened substantially (d = −0.57, p = 0.025). Propensity score matching on age yielded 23 age-balanced pairs (depressive-disorder: 29.7 ± 5.5 years; non-depressed: 29.6 ± 5.5; p = 0.958), but at the cost of 52% of depressive-disorder participants (all those aged >40). In this matched sample, no gene showed a significant group difference (CADM1 p = 0.965; all genes p > 0.13), suggesting that the CADM1 signal in the full sample is at least partially confounded by age. In the quality-based sensitivity analysis excluding samples with 260/230 < 1.0 (48 non-depressed, 44 depressive-disorder), the CADM1 signal strengthened from p = 0.049 to p = 0.031 (d = −0.26), and RAPH1 reached significance (p = 0.041, d = −0.16). Principal component analysis on the quality-controlled data showed a persistent PC2 group difference (p = 0.006), with PC3 also reaching significance (p = 0.040).

A pre-specified diagnostic-severity-restricted sensitivity analysis was performed to address the possibility that pooling major depressive disorder with dysthymia and adjustment disorder with depressed features had diluted a major-depressive-disorder-specific signal. Eight clinical participants had BDI-II scores in the mild range (14–19), most plausibly capturing the diagnostically milder sub-stratum (dysthymia or adjustment disorder); 40 had BDI-II ≥ 20 (moderate-to-severe depression, most plausibly mapping onto major depressive disorder). When the clinical group was restricted to participants with BDI-II ≥ 20, the CADM1 group difference relative to non-depressed controls strengthened rather than weakened (Mann-Whitney p = 0.029, Cohen’s d = −0.28). Further restricting to the severe stratum (BDI-II ≥ 29; n = 31), corresponding most closely to the symptom profile of a current major depressive episode, strengthened the CADM1 signal still further (p = 0.014, d = −0.34, Cliff’s δ = 0.33). The CADM1 signal therefore strengthened monotonically as the case definition was restricted to more severely depressed participants. However, age-adjusted analysis of variance within the severe stratum rendered the CADM1 effect non-significant (p = 0.43), confirming that severity stratification did not rescue the signal from the age confound. RAPH1 also reached uncorrected significance in this subgroup (p = 0.026, d = −0.20). The dimensional CADM1-versus-BDI-II correlation in this subset was −0.297 (p = 0.005). These findings argue against diagnostic heterogeneity as the primary driver of the CADM1 signal: the signal was not generated by, and indeed appears slightly attenuated by, inclusion of the diagnostically milder sub-stratum. However, given the small size of the mild sub-stratum (n = 8), this sensitivity result cannot be regarded as definitively excluding diagnostic-heterogeneity confounding, and replication in a sample stratified by formally recorded diagnostic subcategory is required.

This pattern, in which signals strengthen monotonically with improved data quality and with progressive diagnostic-severity restriction but disappear after age matching, indicates that the CADM1 finding reflects a mixture of biological variation and age confounding that cannot be fully disentangled in this sample.

### 3.11 *Post-hoc* power

*Post-hoc* power for individual-gene comparisons was low (5-19%), reflecting the modest effect sizes typical of peripheral blood gene expression studies in depression. Achieving 80% power at the observed effect sizes would require approximately 300–1200 participants per group for individual genes, with smaller samples required for the PC2 composite (estimated 80% power: n ≈ 95 per group, comparable to the current sample, which yielded 83% power for this composite specifically).

## 4 Discussion

### 4.1 Principal findings

This pilot study is, to our knowledge, the first to examine peripheral blood gene expression in depression within a United Arab Emirates population. Five observations emerged, all of which must be interpreted as hypothesis-generating rather than as evidence of established biomarker properties, given the substantial age confound, the small sample (under 50 per group), and the absence of multiple-testing-corrected significance for any individual gene. First, CADM1 showed an uncorrected group difference (p = 0.049) that did not survive Benjamini-Hochberg correction (adjusted p = 0.178), strengthened after quality-based filtering (p = 0.031) and monotonically across BDI-II severity strata (moderate-severe p = 0.029; severe p = 0.014), but attenuated after age adjustment (ANCOVA p = 0.378) and disappeared after propensity matching (p = 0.965). Second, a dimensional analysis showed that CADM1 expression correlated with depression severity (BDI-II ρ = −0.280, uncorrected p = 0.005; Benjamini-Hochberg-adjusted p = 0.076), though this full-sample correlation is itself susceptible to the age gradient. Third, principal component analysis identified a multi-gene expression signature (PC2) showing an unadjusted difference between groups (p = 0.006), though MANCOVA attenuated this to p = 0.156. Fourth, exploratory uncorrected subgroup analyses suggested sex-specific patterns, with CADM1 in males and KIAA1539 in females. Fifth, the diagnostic-discrimination performance of CADM1 was poor (AUC = 0.616), well below any clinically useful threshold, and the gene panel added no meaningful discrimination beyond age and sex alone.

### 4.2 CADM1 in context

CADM1 encodes SynCAM1, a synaptic cell adhesion molecule essential for synapse formation and maintenance (30). Altered CADM1 expression has been documented in autism (18) and in CADM1-knockout mouse models showing impaired social and emotional behaviours (17). CADM1 variants have also been associated with body mass index and metabolic traits, conditions frequently comorbid with depression (31). The finding that CADM1 upregulation appeared more pronounced in males (d = −0.40) than females (d = −0.02) is descriptively consistent with sex differences in depression-related transcriptional biology (32), but as an exploratory, uncorrected subgroup observation it cannot be regarded as evidence of a sex-specific effect. The signal among Emiratis (p = 0.029) but not expatriates (p = 0.376) is similarly exploratory and may simply reflect the relatively small subgroup sizes; firm conclusions about ancestry-related modification cannot be drawn here.

### 4.3 Sex-specific patterns

The emergence of KIAA1539 as significant in females (p = 0.010) while CADM1 was significant in males suggests sex-specific molecular pathways may underlie peripheral expression changes in DD. Labonté et al. demonstrated sex-specific transcriptional signatures in post-mortem brains of depressed individuals, with largely non-overlapping gene sets between males and females (32). Our peripheral blood findings, while exploratory, are consistent with this emerging picture and warrant sex-stratified designs in future biomarker studies.

### 4.4 Diagnostic heterogeneity

The clinical group combined major depressive disorder, dysthymia, and adjustment disorder with depressed features, the same pooled construct used in the cohorts in which this candidate panel was originally derived (13, 14), and the diagnostic resolution typically available in primary-care screening contexts. Whether the inclusion of diagnostically milder sub-strata might dilute a major-depressive-disorder-specific signal was examined directly through two pre-specified severity-stratified sensitivity analyses using BDI-II thresholds as a proxy for diagnostic gradient.

The CADM1 group difference strengthened monotonically as the case definition was restricted: full depressive-disorder group p = 0.049 (d = −0.23); moderate-severe stratum (BDI-II ≥ 20, n = 40) p = 0.029 (d = −0.28); severe stratum (BDI-II ≥ 29, n = 31) p = 0.014 (d = −0.34). The opposite pattern would be expected if pooling were diluting a depression-specific signal. The data are therefore more consistent with CADM1 expression being associated with the depressive-disorder spectrum than with diagnostic heterogeneity driving the observed group difference.

This finding does not, however, resolve the age confound: the severe stratum retained the 13-year mean age difference of the full sample, and age-adjusted analysis within this stratum was non-significant (p = 0.43). Pooling and age operate as independent constraints on interpretation, and severity stratification addresses the first but not the second.

Two qualifications remain. BDI-II severity is a continuous symptom-burden measure and does not substitute for formally recorded ICD-10 subcategory, so replication in samples stratified by formal diagnosis is required to confirm the pattern. The mild sub-stratum was also small (n = 8), which limits resolution at the lower end of the severity range.

### 4.5 Multi-gene expression patterns

Principal component analysis identified a composite expression pattern (PC2) that distinguished groups more effectively than individual genes. PC1 (90.3% of variance) likely reflects shared technical variation, underscoring the importance of sample-level quality control in quantitative real-time PCR studies (33). The PC2 group difference was attenuated by age adjustment (p = 0.13), so this finding cannot be interpreted as definitive evidence of depression-related changes and requires age-matched replication.

### 4.6 Comparison with prior literature

The largely null individual-gene results in this study contrast with the findings of Pajer et al., who reported significant differential expression of these candidates in adolescents with early-onset depressive disorders (14). Several factors may explain this discrepancy. First, our study was substantially underpowered (5–19% power for individual genes) compared to the effect sizes typically reported in transcriptomic studies. Second, the age confound means we cannot disentangle depression-related from age-related expression changes. Third, unmeasured confounders, particularly medication status, which is known to alter blood gene expression (34, 35), may have introduced noise. Fourth, population differences between the original North American cohorts and our United Arab Emirates sample may exist. Notably, larger transcriptomic studies such as Jansen et al. (n > 18,000) (36) and Leday et al. (12) have demonstrated that peripheral gene expression effects in depressive disorders are typically small (d < 0.3), requiring samples of hundreds to thousands to detect reliably.

### 4.7 The age confound

A central interpretive challenge is that the 13.3-year mean age difference between groups is a plausible alternative explanation for all observed findings (37). Multiple convergent analyses underscore this: ANCOVA attenuated CADM1 (p = 0.378) and PC2 (p = 0.130); MANCOVA rendered the seven-gene profile non-significant (p = 0.156 versus MANOVA p = 0.005); propensity score matching on age eliminated all gene expression differences (CADM1 p = 0.965); and logistic regression showed that age alone (AUC = 0.848) classified groups nearly as well as age plus genes (AUC = 0.854). Against this, the CADM1 signal strengthened when RNA quality outliers were removed (p = 0.031), the dimensional correlation with BDI-II (ρ = −0.280, p = 0.005) persisted in the full sample, and the diagnostic-severity-restricted analyses (moderate-severe p = 0.029; severe p = 0.014) confirmed that the signal was not generated by the diagnostically milder sub-strata. However, the severe stratum carried the same age confound as the full clinical group (mean age 36.4 years), and age-adjusted analysis within this stratum was non-significant (p = 0.43). Taken together, the CADM1 finding most likely reflects a mixture of potential depression-related expression differences and age confounding that cannot be disentangled in this sample. Only an adequately powered, strictly age-matched replication can resolve this ambiguity. The reader is cautioned that the post-matching null result is the more rigorous of the two CADM1 estimates, and is the appropriate basis for any forward-looking interpretation.

### 4.8 Diagnostic discrimination performance

The receiver operating characteristic analysis was pre-specified for the candidate panel as a whole and is reported transparently here, but the resulting AUC values (CADM1 0.616, RAPH1 0.615) indicate no clinically useful diagnostic discrimination. The conventional thresholds for biomarker translation are AUC ≥ 0.80 for acceptable discrimination and AUC ≥ 0.90 for excellent discrimination; values near 0.6 are routinely regarded as falling within the descriptive boundary between chance classification and weak signal. Furthermore, the lower bound of the CADM1 95% confidence interval (0.500) touches the chance-classification line, and the logistic regression analysis showed that adding the entire gene panel to a model containing age and sex improved AUC by only 0.006. We therefore explicitly state, and reiterate in the Conclusions and Limitations, that the present study provides no evidence supporting the use of CADM1, RAPH1, or any individual gene from this panel, or the panel as a whole, as a peripheral diagnostic test for depressive disorders. The biological interest of CADM1 is hypothesis-generating only.

### 4.9 Limitations

Several limitations must be acknowledged. First, the sample was small (n < 50 per group; total n = 97 after exclusions). For the effect sizes typical of psychiatric peripheral gene expression studies (d < 0.3), the study was substantially underpowered (*post-hoc* power 5–19% for individual genes), though the PC2 finding had adequate power (83%). Detecting effects of this magnitude reliably requires samples of several hundred per group, which this pilot study could not provide. This is the principal reason any individual-gene finding here must be regarded as hypothesis-generating rather than confirmatory.

Second, the 13.3-year mean age difference between groups is a substantial confound that cannot be fully resolved statistically, as there is limited overlap in the upper age range, and which propensity matching showed eliminates the CADM1 signal entirely. This is the dominant interpretive constraint on the present findings.

Third, several important variables that are known or plausible modifiers of peripheral blood gene expression were not recorded in the original dataset and therefore could not be statistically controlled. These include medication status (antidepressant exposure is known to alter peripheral blood gene expression (34, 35)), body mass index (relevant to CADM1 expression given its association with metabolic traits (31)), smoking status, duration of illness, comorbid medical conditions, fasting status at the time of blood draw, time of day of phlebotomy, and the specific extraction batch in which each sample was processed. Blood samples were collected at varying times of day depending on participant availability, and RNA extraction was performed across multiple processing batches, introducing potential circadian (38) and batch effects that could not be assessed or corrected *post-hoc*. Each of these unmeasured variables represents a plausible alternative explanation for at least part of the observed CADM1 pattern, and future studies should record them as standard.

Fourth, GAPDH was the sole reference gene used for normalisation, with an inter-sample coefficient of variation of 8.0% that exceeds the MIQE-recommended 5% threshold (33, 39). Single-housekeeping-gene normalisation is increasingly regarded as suboptimal for peripheral blood transcript quantification, because no single reference gene is invariant across all biological and technical conditions. Future studies should employ geometric averaging across multiple validated reference genes selected by tools such as geNorm or NormFinder, as proposed by Vandesompele et al. (40), and pre-validated for the depressive-disorder context (40). The use of GAPDH alone here therefore adds an additional layer of normalisation uncertainty over and above the small-sample and age-confound limitations.

Fifth, primer sequences for KIAA1539 and the reference gene GAPDH could not be retrieved from the original laboratory documentation. While all reactions used validated commercial assays under standardised conditions, this gap in reproducibility documentation is a substantive limitation: external laboratories cannot replicate these specific assays without re-deriving and re-validating primers for these two targets. This is explicitly framed as a non-trivial reproducibility constraint of the present study rather than a minor reporting omission, and we recommend that any replication work re-derive and report complete primer sequences for all targets and reference genes.

Sixth, RNA integrity numbers from Bioanalyzer or TapeStation platforms were not available; only NanoDrop spectrophotometry was performed. While all samples met the 260/280 ≥ 1.80 standard, integrity numbers would have provided a more comprehensive quality assessment, particularly for transcripts vulnerable to partial RNA degradation. The lower 260/230 ratios in the depressive-disorder group, although still within an acceptable range for the majority of samples, leave open the possibility of residual matrix-effect or carry-over contamination, partially mitigated by our quality-based sensitivity analysis.

Seventh, the broad diagnostic grouping (major depressive disorder, dysthymia, adjustment disorder with depressed features) and the absence of a discrete diagnostic subcategory field in the analytic dataset meant that diagnostic-heterogeneity assessment had to be conducted using BDI-II severity as a clinical proxy. Although the severity-restricted sensitivity analysis was reassuring, formally recorded diagnostic subcategory should be a standard data-collection element in replication studies.

Finally, the diagnostic discrimination performance documented here (CADM1 AUC = 0.616, RAPH1 AUC = 0.615, marginal improvement over age-and-sex alone of ΔAUC = 0.006) is well below any clinically useful threshold and the present findings should not be cited as supporting CADM1 or any panel-derived biomarker as a candidate for clinical translation. The biological interest of CADM1 in depression, supported by independent mechanistic evidence (17, 18, 30), is unaffected by this point; what we cannot support, on the present data, is its utility as a peripheral diagnostic test.

### 4.10 Strengths

Several features of the present study merit emphasis. To our knowledge, this is the first peripheral blood gene-expression dataset in depressive disorders from a United Arab Emirates population, and among the few such datasets from the Middle East and North Africa region. The study therefore provides baseline molecular data from a population that remains substantially underrepresented in psychiatric transcriptomics.

The analytical strategy was deliberately convergent, particularly for a study of this size. The principal CADM1 signal was examined using Mann–Whitney U testing, age-adjusted analysis of covariance, multivariate analysis of covariance across the full seven-gene panel, robust bootstrap regression, propensity-score matching, ten-fold cross-validated logistic regression, dimensional Spearman correlation with depression severity, two pre-specified severity-stratified sensitivity analyses, and an RNA-quality-restricted sensitivity analysis. These approaches yielded a consistent overall interpretation, allowing the stability of the candidate signal to be assessed across analytic specifications rather than inferred from a single model. Multiple testing was controlled using Benjamini–Hochberg false discovery rate correction, and key analyses were pre-specified.

Importantly, negative and potentially confounded findings are reported transparently. The propensity-matched null result for CADM1 is presented in the main findings as the more rigorous estimate, rather than relegated to supplementary material. Similarly, receiver operating characteristic performance is interpreted against the conventional threshold for acceptable discrimination, rather than framed merely in comparison with chance.

Technical reproducibility is strengthened by triplicate measurement of every gene–sample combination, reporting of intra-assay variability, quantitative documentation of the quality-control criteria underlying the single sample exclusion, and open deposition of the full analytic dataset. Together, these measures allow the analyses reported here to be independently reproduced.

### 4.11 Future directions

Several directions merit pursuit beyond the methodological improvements noted above. First, future studies should recruit substantially larger samples (≥ 300 per group) with strict age-matching within narrow bands (for example, 5-year strata), comprehensive clinical metadata (medication status, duration of illness, body mass index, smoking status, comorbidities, formally recorded diagnostic subcategory), and standardised blood collection protocols to minimise circadian and batch effects. RNA quality should be assessed using RNA integrity numbers (≥ 7.0) alongside spectrophotometry, and normalisation should employ geometric averaging across multiple validated reference genes (40).

Second, the field has moved beyond single-gene and candidate-gene approaches toward hypothesis-free discovery using whole-transcriptome RNA sequencing. Large consortium studies such as Jansen et al. (36) and Leday et al. (12) have demonstrated that individual peripheral gene expression effects in depression are typically small, requiring thousands of participants for reliable detection. Future validation of any signal of interest should therefore be embedded within broader transcriptomic profiling rather than isolated quantitative real-time PCR replication, allowing simultaneous discovery of novel candidates and contextualisation of CADM1 within co-expression networks.

Third, recent work has shown that multi-omics integration, combining transcriptomics with DNA methylation and microRNA profiling, substantially improves classification accuracy for depression compared with any single molecular layer alone, with sex-specific analyses further enhancing predictive performance (41, 42). Given the exploratory sex-specific patterns observed in this study, sex-stratified multi-omic designs would be particularly informative in this population.

Fourth, the underrepresentation of Middle Eastern and North African populations in global genomic databases remains a significant barrier to precision psychiatry in the region (43, 44). Cultural, environmental, and genetic factors, including high consanguinity rates in some Gulf populations, may influence both disease presentation and molecular profiles in ways that cannot be inferred from studies conducted in European or North American cohorts. The present study adds to a small but growing body of region-specific molecular psychiatry data and underscores the need for multi-centre collaborations across Gulf Cooperation Council countries to achieve the sample sizes necessary for adequately powered molecular biomarker studies.

Finally, CADM1 warrants mechanistic investigation beyond peripheral blood expression. As a synaptic cell adhesion molecule critical for synapse formation, its potential role in depression may be better captured through parallel assessment in central nervous system tissue, cerebrospinal fluid, or through integration with neuroimaging data linking peripheral expression to brain structural or functional changes (45). The convergence of peripheral blood biomarkers with brain-level data represents a promising frontier for translational depression research.

## 5 Conclusions

This pilot study provides the first peripheral blood gene expression data for depression in a United Arab Emirates population. Within the limits imposed by the small sample (n < 50 per group), the 13-year mean age difference between groups, multiple unmeasured confounders (medication status, body mass index, smoking, duration of illness, circadian and batch effects), single-housekeeping-gene normalisation, and the absence of multiple-testing-corrected individual-gene significance, the data support only the following conclusions. First, CADM1 showed an uncorrected, modest group-level signal (p = 0.049, BH-adjusted p = 0.178) and an uncorrected dimensional correlation with depression severity (BDI-II ρ = −0.280, p = 0.005), with the signal strengthening monotonically across BDI-II severity strata (moderate-severe p = 0.029; severe p = 0.014) but eliminated under propensity-score age matching (p = 0.965). Second, the diagnostic discrimination performance of CADM1 in this sample (AUC = 0.616) and of the entire seven-gene panel beyond age and sex (ΔAUC = 0.006) is well below any clinically useful threshold; no individual gene or composite from this panel can be regarded as a peripheral diagnostic biomarker on the basis of these data. Third, exploratory subgroup patterns (CADM1 in males, KIAA1539 in females) and the multi-gene PC2 signature are hypothesis-generating only, requiring confirmation in age-matched, multi-omic, sex-stratified designs. CADM1 should therefore be regarded as a biologically plausible candidate worth further mechanistic investigation, not as a clinically translatable biomarker. The principal contribution of this pilot is regional and methodological: it provides baseline peripheral expression estimates for a previously unstudied population, identifies the specific design requirements for adequately powered replication, and quantifies how far the current candidate-gene approach is from clinical translation in this setting.

## Data Availability

The datasets presented in this study can be found in online repositories. The names of the repository/repositories and accession number(s) can be found below: https://doi.org/10.17605/OSF.IO/7KXAZ, 10.17605/OSF.IO/7KXAZ.

## Glossary

ANCOVA: Analysis of covariance
AUC: Area under the curve
BDI-II: Beck Depression Inventory-II
BH: Benjamini-Hochberg
BMI: Body mass index
cDNA: Complementary deoxyribonucleic acid
CI: Confidence interval
CT: Cycle threshold
CV: Coefficient of variation
DD: Depressive disorders
GAPDH: Glyceraldehyde 3-phosphate dehydrogenase
ICD-10: International Classification of Diseases, 10th revision
IQR: Interquartile range
MANCOVA: Multivariate analysis of covariance
MANOVA: Multivariate analysis of variance
MDD: Major depressive disorder
ND: Non-depressed
PCA: Principal component analysis
PHQ-9: Patient Health Questionnaire-9
qRT-PCR: Quantitative real-time polymerase chain reaction
RIN: RNA Integrity Number
RNA: Ribonucleic acid
RT: Reverse transcription
ROC: Receiver operating characteristic
SD: Standard deviation
UAE: United Arab Emirates
