# Supplementary material for: Peripheral blood CADM1 expression and multi-gene signatures in depressive disorders: a pilot case-control study from the United Arab Emirates
Source: Front Psychiatry. 2026 Jul 1;17:1850124. doi: 10.3389/fpsyt.2026.1850124 (PMC13371162; doi:10.3389/fpsyt.2026.1850124)
Supplement: Supplementary file 1 [file SupplementaryFile1.docx]

Supplementary Material

# Table of Contents

Table S1. Gene primer details

Table S2. ANCOVA comparisons of ΔCT between groups, controlling for age

Table S3. Individual depression severity scores

Table S4. RNA quality metrics for all samples

Table S5. Triplicate CT standard errors for all sample-gene combinations

Table S6. Intra-assay triplicate variability by gene

Table S7. Robust bootstrap regression results for all genes

Table S8. PCA of ΔCT values: variance and gene loadings

Table S9. Sex-stratified and nationality-stratified Mann-Whitney U results for all genes

Table S10. Logistic regression classification of DD vs ND

Table S11. Logistic regression standardised coefficients (genes-only model)

Table S12. Spearman correlations between gene expression (ΔCT) and depression severity

Table S13. Within-DD gene expression-depression severity correlations

Table S14. Severity-stratified Mann-Whitney U comparisons of ΔCT between DD and DD control groups for all seven candidate genes

# Supplementary Table S1. Gene primer details

| Gene | Forward Primer (5'→3') | Reverse Primer (5'→3') | Amplicon (bp) |
| --- | --- | --- | --- |
| ADCY3 | CGAGAACGTCAGCATCCTCTTT | GGCACTGCAGGCAGAAGAC | 66 |
| DGKA | ACCCTTCCCATGCAAATTGA | CTTGTGGGTGATCTTGATTGTACAG | 69 |
| FAM46A | CTCACGCTCAAGGAAGCTTATGT | GGATATAAGACTCCATCGGTCAGAGT | 75 |
| CADM1 | AGCAAGCCAGCCACGACTAT | CGATTTGCCTTTTAGCTCTGTGT | 60 |
| MARCKS | CCTCTTGGATCTGTTGAGTTTCTTT | ATTCTCCTGTCCGTTCGCTTT | 148 |
| RAPH1 | AGCAAGGCCAGAATGGAGTCTAT | GTGGTGAAGGCTGTGAAGCA | 109 |

**Note**. Primers from TaqMan Array Tools (Thermo Fisher Scientific). GAPDH served as endogenous control. Primer sequences for KIAA1539 and GAPDH were not recorded by the original laboratory.

## Supplementary Table S2. ANCOVA comparisons of ΔCT between groups, controlling for age.

| Gene | Effect | SS | df | MS | F | p | η²_p |
| --- | --- | --- | --- | --- | --- | --- | --- |
| ADCY3 | Group | 0.876 | 1 | 0.876 | 0.392 | 0.533 | 0.004 |
|  | Age | 1.261 | 1 | 1.261 | 0.564 | 0.455 | 0.006 |
| CADM1 | Group | 1.883 | 1 | 1.883 | 0.658 | 0.378 | 0.007 |
|  | Age | 0.035 | 1 | 0.035 | 0.012 | 0.912 | 0.000 |
| DGKA | Group | 0.122 | 1 | 0.122 | 0.056 | 0.814 | 0.001 |
|  | Age | 1.999 | 1 | 1.999 | 0.917 | 0.341 | 0.010 |
| FAM46A | Group | 0.006 | 1 | 0.006 | 0.003 | 0.959 | 0.000 |
|  | Age | 2.036 | 1 | 2.036 | 0.944 | 0.334 | 0.010 |
| RAPH1 | Group | 1.168 | 1 | 1.168 | 0.470 | 0.495 | 0.005 |
|  | Age | 0.112 | 1 | 0.112 | 0.045 | 0.832 | 0.000 |
| KIAA1539 | Group | 0.654 | 1 | 0.654 | 0.291 | 0.591 | 0.003 |
|  | Age | 0.985 | 1 | 0.985 | 0.438 | 0.510 | 0.005 |
| MARCKS | Group | 0.503 | 1 | 0.503 | 0.254 | 0.615 | 0.003 |
|  | Age | 0.037 | 1 | 0.037 | 0.019 | 0.892 | 0.000 |

**Note**. ANCOVA: ΔCT ~ Group + Age. N = 49 ND, 48 DD. No group effect reached significance. DD = depressive disorder.

## Supplementary Table S3. Individual depression severity scores.

| Sample | Group | Age | Gender | Nationality | BDI-II | PHQ-9 |
| --- | --- | --- | --- | --- | --- | --- |
| 1 | DD | 37 | Male | Emirati | 54 | 12 |
| 2 | DD | 37 | Male | Expatriate | 30 | 15 |
| 3 | DD | 32 | Female | Emirati | 26 | 18 |
| 4 | DD | 65 | Male | Expatriate | 17 | 12 |
| 5 | DD | 40 | Male | Expatriate | 29 | 14 |
| 6 | DD | 33 | Male | Expatriate | 39 | 18 |
| 7 | DD | 27 | Female | Emirati | 46 | 20 |
| 8 | DD | 35 | Male | Emirati | 32 | 15 |
| 9 | DD | 26 | Male | Emirati | 16 | 9 |
| 10 | DD | 39 | Female | Emirati | 30 | 21 |
| 11 | DD | 36 | Male | Emirati | 55 | 25 |
| 12 | DD | 28 | Male | Expatriate | 40 | 17 |
| 13 | DD | 29 | Female | Emirati | 39 | 14 |
| 14 | DD | 32 | Male | Emirati | 28 | 14 |
| 15 | DD | 37 | Female | Expatriate | 31 | 14 |
| 17 | DD | 40 | Male | Expatriate | 32 | 14 |
| 18 | DD | 60 | Male | Expatriate | 18 | 11 |
| 19 | DD | 36 | Female | Expatriate | 17 | 5 |
| 20 | DD | 38 | Female | Emirati | 30 | 10 |
| 21 | DD | 52 | Female | Expatriate | 27 | 14 |
| 22 | DD | 55 | Female | Expatriate | 36 | 15 |
| 23 | DD | 48 | Male | Expatriate | 31 | 13 |
| 24 | DD | 42 | Female | Expatriate | 32 | 13 |
| 25 | DD | 45 | Male | Emirati | 55 | 24 |
| 26 | DD | 38 | Female | Emirati | 31 | 14 |
| 27 | DD | 49 | Female | Expatriate | 16 | 5 |
| 28 | DD | 40 | Male | Expatriate | 48 | 20 |
| 29 | DD | 46 | Female | Emirati | 18 | 7 |
| 30 | DD | 20 | Female | Expatriate | 26 | 15 |
| 31 | DD | 60 | Female | Emirati | 25 | 13 |
| 32 | DD | 50 | Female | Expatriate | 16 | 8 |
| 33 | DD | 38 | Female | Expatriate | 22 | 11 |
| 34 | DD | 51 | Female | Emirati | 46 | 19 |
| 35 | DD | 32 | Female | Emirati | 36 | 16 |
| 36 | DD | 48 | Male | Expatriate | 30 | 13 |
| 37 | DD | 20 | Female | Expatriate | 37 | 14 |
| 38 | DD | 39 | Female | Emirati | 40 | 19 |
| 39 | DD | 54 | Female | Expatriate | 50 | 18 |
| 40 | DD | 30 | Female | Expatriate | 36 | 16 |
| 41 | DD | 22 | Female | Emirati | 40 | 18 |
| 42 | DD | 24 | Male | Emirati | 30 | 15 |
| 43 | DD | 31 | Male | Expatriate | 14 | 10 |
| 44 | DD | 23 | Male | Emirati | 43 | 24 |
| 45 | DD | 30 | Male | Emirati | 34 | 14 |
| 46 | DD | 31 | Male | Expatriate | 25 | 14 |
| 47 | DD | 43 | Male | Emirati | 42 | 15 |
| 48 | DD | 41 | Male | Expatriate | 26 | 15 |
| 49 | DD | 38 | Male | Emirati | 28 | 15 |
| 50 | DD | 27 | Male | Emirati | 43 | 20 |
| 51 | ND | 28 | Female | Emirati | 2 | 1 |
| 52 | ND | 30 | Female | Expatriate | 8 | 3 |
| 53 | ND | 36 | Female | Expatriate | 9 | 3 |
| 54 | ND | 23 | Female | Expatriate | 3 | 0 |
| 55 | ND | 25 | Female | Emirati | 7 | 4 |
| 56 | ND | 33 | Female | Expatriate | 8 | 2 |
| 57 | ND | 30 | Male | Expatriate | 10 | 0 |
| 58 | ND | 39 | Female | Expatriate | 6 | 2 |
| 59 | ND | 31 | Female | Expatriate | 2 | 1 |
| 60 | ND | 23 | Female | Expatriate | 3 | 2 |
| 61 | ND | 35 | Female | Emirati | 9 | 4 |
| 62 | ND | 18 | Male | Emirati | 6 | 4 |
| 63 | ND | 19 | Male | Emirati | 2 | 2 |
| 64 | ND | 18 | Male | Emirati | 7 | 4 |
| 65 | ND | 20 | Male | Emirati | 0 | 0 |
| 66 | ND | 27 | Female | Emirati | 0 | 0 |
| 67 | ND | 32 | Female | Emirati | 3 | 2 |
| 68 | ND | 31 | Female | Expatriate | 1 | 2 |
| 69 | ND | 18 | Male | Emirati | 6 | 4 |
| 70 | ND | 22 | Female | Expatriate | 9 | 3 |
| 71 | ND | 32 | Male | Expatriate | 0 | 0 |
| 72 | ND | 35 | Male | Expatriate | 10 | 4 |
| 73 | ND | 29 | Male | Expatriate | 9 | 4 |
| 74 | ND | 26 | Male | Expatriate | 9 | 4 |
| 75 | ND | 23 | Female | Expatriate | 1 | 1 |
| 76 | ND | 18 | Male | Emirati | 4 | 3 |
| 77 | ND | 20 | Male | Emirati | 8 | 4 |
| 78 | ND | 23 | Female | Expatriate | 11 | 4 |
| 79 | ND | 24 | Female | Expatriate | 5 | 4 |
| 80 | ND | 27 | Female | Emirati | 7 | 0 |
| 82 | ND | 23 | Female | Emirati | 9 | 4 |
| 83 | ND | 39 | Female | Emirati | 2 | 0 |
| 84 | ND | 32 | Female | Expatriate | 7 | 3 |
| 85 | ND | 26 | Female | Expatriate | 3 | 1 |
| 86 | ND | 24 | Female | Expatriate | 2 | 1 |
| 87 | ND | 28 | Male | Expatriate | 4 | 0 |
| 88 | ND | 19 | Male | Expatriate | 9 | 4 |
| 89 | ND | 18 | Male | Emirati | 0 | 0 |
| 90 | ND | 18 | Male | Emirati | 2 | 2 |
| 91 | ND | 19 | Male | Emirati | 5 | 4 |
| 92 | ND | 22 | Male | Emirati | 6 | 4 |
| 93 | ND | 20 | Male | Emirati | 10 | 4 |
| 94 | ND | 23 | Male | Expatriate | 11 | 4 |
| 95 | ND | 18 | Male | Expatriate | 4 | 4 |
| 96 | ND | 21 | Male | Expatriate | 9 | 4 |
| 97 | ND | 24 | Female | Expatriate | 4 | 3 |
| 98 | ND | 26 | Female | Expatriate | 1 | 0 |
| 99 | ND | 18 | Male | Expatriate | 12 | 5 |
| 100 | ND | 23 | Male | Expatriate | 2 | 2 |

**Note**. BDI-II: Beck Depression Inventory-II (range 0–63). PHQ-9: Patient Health Questionnaire-9 (range 0–27). DD = clinical group (see Methods for diagnostic composition). ND = non-depressed control. DD = depressive disorder; ND = non-depressed.

## Supplementary Table S4. RNA quality metrics for all samples.

| Sample | Group | Concentration (ng/μL) | 260/280 | 260/230 | QC Flag |
| --- | --- | --- | --- | --- | --- |
| Control 51 | Control | Missing | Missing | Missing | Missing NanoDrop data |
| Control 52 | Control | 96.8 | 2.06 | 1.78 |  |
| Control 53 | Control | 92.2 | 2.03 | 1.94 |  |
| Control 54 | Control | 103.0 | 2.03 | 1.80 |  |
| Control 55 | Control | 103.6 | 2.05 | 1.92 |  |
| Control 56 | Control | 65.6 | 2.04 | 1.37 |  |
| Control 57 | Control | 85.0 | 2.06 | 2.01 |  |
| Control 58 | Control | 59.2 | 2.03 | 1.59 |  |
| Control 59 | Control | 79.5 | 2.06 | 1.85 |  |
| Control 60 | Control | 62.8 | 2.01 | 0.77 | 260/230 < 1.0 |
| Control 61 | Control | 102.6 | 2.03 | 1.49 |  |
| Control 62 | Control | 109.0 | 2.04 | 2.16 |  |
| Control 63 | Control | 169.8 | 2.05 | 2.12 |  |
| Control 64 | Control | 134.5 | 2.03 | 2.14 |  |
| Control 65 | Control | 205.8 | 2.07 | 2.15 |  |
| Control 66 | Control | 169.4 | 2.08 | 1.60 |  |
| Control 67 | Control | 210.4 | 2.04 | 1.98 |  |
| Control 68 | Control | 134.9 | 2.05 | 2.13 |  |
| Control 69 | Control | 282.7 | 2.06 | 2.12 |  |
| Control 70 | Control | 98.4 | 2.01 | 2.09 |  |
| Control 71 | Control | 148.8 | 2.03 | 2.08 |  |
| Control 72 | Control | 164.1 | 2.05 | 1.93 |  |
| Control 73 | Control | 113.9 | 2.06 | 2.04 |  |
| Control 74 | Control | 208.3 | 2.04 | 2.07 |  |
| Control 75 | Control | 97.4 | 2.06 | 2.01 |  |
| Control 76 | Control | 143.3 | 2.01 | 2.12 |  |
| Control 77 | Control | 155.4 | 2.02 | 1.85 |  |
| Control 78 | Control | 193.5 | 2.04 | 2.06 |  |
| Control 79 | Control | 114.4 | 2.03 | 1.94 |  |
| Control 80 | Control | 157.4 | 2.03 | 2.11 |  |
| Control 81 | Control | 41.3 | 2.04 | 1.73 | Not in analytic sample (excluded pre-analysis) |
| Control 82 | Control | 74.3 | 1.99 | 1.99 |  |
| Control 83 | Control | 171.4 | 2.03 | 2.11 |  |
| Control 84 | Control | 76.8 | 2.03 | 1.95 |  |
| Control 85 | Control | 82.4 | 2.05 | 2.12 |  |
| Control 86 | Control | 79.1 | 2.03 | 2.03 |  |
| Control 87 | Control | 153.2 | 2.04 | 2.11 |  |
| Control 88 | Control | 145.7 | 2.02 | 2.05 |  |
| Control 89 | Control | 163.4 | 2.03 | 2.03 |  |
| Control 90 | Control | 126.3 | 2.02 | 1.28 |  |
| Control 91 | Control | 88.2 | 2.05 | 1.48 |  |
| Control 92 | Control | 105.2 | 2.03 | 1.88 |  |
| Control 93 | Control | 127.7 | 2.04 | 1.90 |  |
| Control 94 | Control | 145.0 | 2.05 | 1.80 |  |
| Control 95 | Control | 166.7 | 2.06 | 2.09 |  |
| Control 96 | Control | 197.0 | 2.06 | 2.10 |  |
| Control 97 | Control | 211.1 | 2.06 | 1.95 |  |
| Control 98 | Control | 75.9 | 2.01 | 1.94 |  |
| Control 99 | Control | 229.7 | 2.04 | 2.10 |  |
| Control 100 | Control | 132.9 | 2.04 | 1.87 |  |
| Patient 1 | Patient | 144.8 | 2.07 | 2.16 |  |
| Patient 2 | Patient | 51.1 | 1.99 | 1.82 |  |
| Patient 3 | Patient | 141.4 | 2.06 | 2.09 |  |
| Patient 4 | Patient | 117.4 | 2.02 | 1.85 |  |
| Patient 5 | Patient | 107.2 | 2.07 | 1.89 |  |
| Patient 6 | Patient | 281.0 | 2.03 | 2.13 |  |
| Patient 7 | Patient | 48.3 | 2.00 | 1.73 |  |
| Patient 8 | Patient | 121.2 | 2.02 | 2.16 |  |
| Patient 9 | Patient | 37.8 | 2.06 | 0.51 | 260/230 < 1.0 |
| Patient 10 | Patient | 498.5 | 2.08 | 2.07 | Excluded (RT failure) |
| Patient 11 | Patient | 105.9 | 2.06 | 1.70 |  |
| Patient 12 | Patient | 171.8 | 2.04 | 1.76 |  |
| Patient 13 | Patient | 158.4 | 2.05 | 2.08 |  |
| Patient 14 | Patient | 175.4 | 2.04 | 2.08 |  |
| Patient 15 | Patient | 87.5 | 2.03 | 2.01 |  |
| Patient 16 | Patient | 18.2 | 1.93 | 0.62 | Not in analytic sample (excluded pre-analysis); 260/230 < 1.0 |
| Patient 17 | Patient | 61.7 | 2.03 | 1.71 |  |
| Patient 18 | Patient | 76.3 | 2.05 | 1.86 |  |
| Patient 19 | Patient | 67.9 | 2.03 | 1.91 |  |
| Patient 20 | Patient | 116.1 | 2.04 | 1.98 |  |
| Patient 21 | Patient | 48.0 | 2.04 | 1.23 |  |
| Patient 22 | Patient | 65.2 | 2.02 | 1.94 |  |
| Patient 23 | Patient | 128.8 | 2.05 | 1.73 |  |
| Patient 24 | Patient | 428.8 | 2.05 | 1.66 |  |
| Patient 25 | Patient | 129.5 | 2.01 | 2.04 |  |
| Patient 26 | Patient | 184.0 | 2.03 | 1.89 |  |
| Patient 27 | Patient | 263.2 | 2.03 | 2.07 |  |
| Patient 28 | Patient | 156.9 | 2.05 | 1.67 |  |
| Patient 29 | Patient | 88.8 | 2.05 | 1.91 |  |
| Patient 30 | Patient | 67.5 | 1.95 | 2.00 |  |
| Patient 31 | Patient | 73.9 | 2.04 | 1.84 |  |
| Patient 32 | Patient | 108.8 | 2.06 | 1.78 |  |
| Patient 33 | Patient | 125.4 | 2.07 | 1.74 |  |
| Patient 34 | Patient | 152.0 | 2.06 | 1.90 |  |
| Patient 35 | Patient | 43.2 | 2.02 | 0.92 | 260/230 < 1.0 |
| Patient 36 | Patient | 105.8 | 2.04 | 0.77 | 260/230 < 1.0 |
| Patient 37 | Patient | 170.5 | 2.06 | 1.78 |  |
| Patient 38 | Patient | 95.6 | 2.04 | 2.14 |  |
| Patient 39 | Patient | 257.5 | 1.98 | 1.69 |  |
| Patient 40 | Patient | 186.9 | 2.01 | 2.05 |  |
| Patient 41 | Patient | 62.7 | 2.02 | 1.58 |  |
| Patient 42 | Patient | 149.5 | 2.05 | 0.48 | 260/230 < 1.0 |
| Patient 43 | Patient | 79.1 | 2.02 | 1.53 |  |
| Patient 44 | Patient | 178.9 | 2.04 | 1.87 |  |
| Patient 45 | Patient | 89.3 | 2.09 | 1.99 |  |
| Patient 46 | Patient | 53.6 | 2.05 | 1.17 |  |
| Patient 47 | Patient | 39.8 | 1.92 | 1.88 |  |
| Patient 48 | Patient | 161.8 | 2.02 | 2.02 |  |
| Patient 49 | Patient | 198.6 | 2.02 | 2.02 |  |
| Patient 50 | Patient | 92.5 | 2.04 | 2.16 |  |

**Note**. NanoDrop spectrophotometry results. 50 patients (1–50) and 50 controls (51–100) were assayed. Patient 16 and Control 81 were excluded from the study pre-analysis (no questionnaire or molecular data available). Control 51 lacked NanoDrop data. Patient 10 was excluded from gene expression analyses for cDNA/RT failure. Final molecular analytic sample: 48 depressive disorder and 49 non-depressed. Sample identifiers are study-specific only and do not correspond to hospital identifiers. Demographic variables are provided only in broad categories.

## Supplementary Table S5. Triplicate CT standard errors for all sample–gene combinations.

| Sample | GAPDH SE | ADCY3 SE | CADM1 SE | DGKA SE | FAM46A SE | KIAA1539 SE | MARCKS SE | RAPH1 SE |
| --- | --- | --- | --- | --- | --- | --- | --- | --- |
| Control 100 | 0.5436 | 0.1169 | 0.0408 | 0.1876 | 0.0160 | 0.2185 | 0.2360 | 0.3281 |
| Control 51 | 0.2685 | 0.1461 | 0.2689 | 0.0704 | 0.0346 | 0.0073 | 0.6205 | 0.1783 |
| Control 52 | 0.0644 | 0.2369 | 0.0051 | 0.3914 | 0.0158 | 0.1987 | 0.3335 | 0.1625 |
| Control 53 | 0.0367 | 0.1785 | 0.2510 | 0.3081 | 0.0390 | 0.0713 | 0.1422 | 0.2065 |
| Control 54 | 0.1834 | 0.0241 | 0.0116 | 0.0969 | 0.2914 | 0.0094 | 0.1673 | 0.1314 |
| Control 55 | 0.0056 | 0.2012 | 0.1929 | 0.1778 | 0.1917 | 0.0080 | 0.4439 | 0.1174 |
| Control 56 | 0.1847 | 0.2209 | 0.0238 | 0.0342 | 0.0136 | 0.5720 | 0.3703 | 0.0088 |
| Control 57 | 0.1599 | 0.0092 | 0.1304 | 0.0359 | 0.2481 | 0.1248 | 0.1477 | 0.0384 |
| Control 58 | 0.1369 | 0.3303 | 0.0684 | 0.1723 | 0.2176 | 0.0365 | Missing | 0.4941 |
| Control 59 | 0.0455 | 0.0465 | 0.0496 | 0.0478 | 0.0131 | 0.0087 | 0.0430 | 0.0027 |
| Control 60 | 0.3160 | 0.2451 | 0.2793 | 0.1582 | 0.2718 | 0.2903 | 0.5613 | 0.3370 |
| Control 61 | 0.0289 | 0.1226 | 0.0654 | 0.0429 | 0.0773 | 0.0202 | 0.0755 | 0.0817 |
| Control 62 | 0.0473 | 0.0600 | 0.0473 | 0.0695 | 0.3560 | 0.4158 | 0.2661 | 0.5834 |
| Control 63 | 0.2147 | 0.3473 | 0.1954 | 0.0482 | 0.1417 | 0.0811 | 0.2363 | 0.2308 |
| Control 64 | 0.1465 | 0.1659 | 0.5565 | 0.3423 | 0.2962 | 0.3195 | 0.3516 | 0.4873 |
| Control 65 | 0.4462 | 0.0965 | 0.0689 | 0.2067 | 0.2819 | 0.3518 | 0.3425 | 0.4278 |
| Control 66 | 0.3150 | 0.1567 | 0.5136 | 0.1016 | 0.6814 | 0.2646 | 0.8075 | 0.8855 |
| Control 67 | 0.0426 | 0.0233 | 0.1252 | 0.0102 | 0.0280 | 0.1020 | 0.1890 | 0.0429 |
| Control 68 | 0.1554 | 0.2550 | 0.4273 | 0.0655 | 0.0434 | 0.0955 | 0.0223 | 0.0199 |
| Control 69 | 0.1001 | 0.0176 | 0.0068 | 0.0131 | 0.0253 | 0.2864 | 0.0360 | 0.0780 |
| Control 70 | 0.0631 | 0.0462 | 0.0133 | 0.0331 | 0.0258 | 0.0136 | 0.0461 | 0.0325 |
| Control 71 | 0.0072 | 0.0012 | 0.0665 | 0.0271 | 0.0335 | 0.0088 | 0.1393 | 0.0582 |
| Control 77 | 0.1152 | 0.1346 | 0.2620 | 0.2330 | 0.1630 | 0.1910 | 0.1886 | 0.0442 |
| Control 78 | 0.2620 | 0.3248 | 0.2335 | 0.7253 | 0.2153 | 0.0524 | 0.0651 | 0.0188 |
| Control 79 | 0.5608 | 0.0812 | 0.1260 | 0.2104 | 0.0700 | 0.1391 | 0.0787 | 0.1513 |
| Control 80 | 0.2386 | 0.0907 | 0.3349 | 0.3097 | 0.3951 | 0.1740 | 0.1187 | 0.1741 |
| Control 82 | 0.3220 | 0.0427 | 0.0760 | 0.8608 | 0.1285 | 0.2306 | 0.3419 | 0.1467 |
| Control 83 | 0.0109 | 0.1454 | 0.0440 | 0.0656 | 0.0531 | 0.0131 | 0.0389 | 0.0266 |
| Control 84 | 0.0335 | 0.0983 | 0.0281 | 0.2595 | 0.0018 | 0.0298 | 0.0373 | 0.1041 |
| Control 85 | 0.0426 | 0.0493 | 0.1203 | 0.0047 | 0.0006 | 0.0289 | 0.0059 | 0.3621 |
| Control 86 | 0.0006 | 0.1469 | 0.0309 | 0.0678 | 0.0346 | 0.0621 | 0.0461 | 0.0773 |
| Control 87 | 0.0580 | 0.1101 | 0.0100 | 0.0553 | 0.0231 | 0.0054 | 0.0100 | 0.0220 |
| Control 88 | 0.0622 | 0.0673 | 0.0054 | 0.0589 | 0.0114 | 0.0109 | 0.0639 | 0.3155 |
| Control 89 | 0.1498 | 0.1666 | 0.4752 | 0.1185 | 0.0335 | 0.3220 | 0.2741 | 0.0671 |
| Control 90 | 0.0289 | 0.0498 | 0.0391 | 0.0204 | 0.0272 | 0.0289 | 0.0553 | 0.0453 |
| Control 91 | 0.1184 | 0.1318 | 0.0998 | 0.1983 | 0.0365 | 0.0153 | 0.1394 | 0.0694 |
| Control 92 | 0.3248 | 0.0203 | 0.1428 | 0.0323 | 0.0318 | 0.0771 | 0.0043 | 0.3976 |
| Control 93 | 0.0512 | 0.0214 | 0.0885 | 0.0234 | 0.0137 | 0.0152 | 0.0406 | 0.0609 |
| Control 94 | 0.0958 | 0.1288 | 0.1498 | Missing | 0.2217 | 0.0480 | 0.4710 | 0.3845 |
| Control 95 | 0.0370 | 0.1422 | 0.0250 | 0.0343 | 0.0048 | 0.0240 | 0.0647 | 0.0814 |
| Control 96 | 0.0953 | 0.3369 | 0.0093 | 0.1172 | 0.0625 | 0.2586 | 0.2572 | 0.1077 |
| Control 97 | 0.0416 | 0.0543 | 0.1294 | 0.1218 | 0.0177 | 0.0360 | 0.0379 | 0.1014 |
| Control 98 | 0.3551 | 0.7128 | 0.0939 | 0.4108 | 0.1957 | 0.0746 | 0.1693 | 0.4122 |
| Control 99 | 0.0407 | 0.0617 | 0.2225 | Missing | 0.1678 | 0.1050 | 0.0162 | 0.0742 |
| Patient 1 | 0.4794 | 0.2194 | 0.6037 | 0.2026 | 0.7649 | 0.0613 | 0.1469 | 0.1942 |
| Patient 10 | 0.0960 | Missing | 0.0676 | 0.1379 | 1.0167 | 0.1093 | 0.1030 | 0.0461 |
| Patient 11 | 0.1123 | 0.2814 | 0.4412 | 0.6776 | 0.4125 | 0.1408 | 0.0075 | 0.5322 |
| Patient 12 | 0.3865 | 0.5870 | 0.0020 | 0.1358 | 0.6452 | 0.2654 | 0.0154 | 0.4669 |
| Patient 13 | 0.1070 | 0.0861 | 0.1418 | 0.0821 | 0.0534 | 0.0016 | 0.1090 | 0.0050 |
| Patient 14 | 0.0126 | 0.0615 | 0.0213 | 0.0588 | 0.0015 | 0.0062 | 0.0215 | 0.0903 |
| Patient 15 | 0.1049 | 0.0080 | 0.0860 | 0.0252 | 0.0007 | 0.0027 | 0.0696 | 0.0757 |
| Patient 17 | 0.0051 | 0.0419 | 0.1681 | 0.2112 | 0.0660 | 0.0666 | 0.0350 | 0.0560 |
| Patient 18 | 0.0143 | 0.0204 | 0.3314 | 0.5578 | 0.5709 | 0.1916 | 0.2476 | 0.3202 |
| Patient 19 | 0.0721 | 0.0676 | 0.0732 | 0.1537 | 0.0476 | Missing | 0.6051 | 0.0204 |
| Patient 2 | 0.0912 | 0.0256 | 0.2644 | 0.4273 | 0.0081 | 0.1821 | 0.5149 | 0.1429 |
| Patient 20 | 0.0028 | 0.0810 | 0.1514 | 0.0400 | 0.0510 | 0.0012 | 0.0049 | 0.0605 |
| Patient 21 | 0.1008 | 0.1856 | 0.0984 | 0.0462 | 0.0752 | 0.3063 | 0.0906 | 0.1826 |
| Patient 22 | 0.1441 | 0.0833 | 0.0452 | 0.0295 | 0.0289 | 0.0727 | 0.0294 | 0.0313 |
| Patient 23 | 0.0311 | 0.0442 | 0.9117 | 0.0026 | 0.1740 | 0.2233 | 0.1530 | 0.3177 |
| Patient 24 | 0.0681 | 0.0176 | 0.0993 | 0.0147 | 0.2950 | 0.0307 | 0.1655 | 0.2837 |
| Patient 25 | 0.0299 | 0.0454 | 0.0098 | 0.0168 | 0.1122 | 0.0668 | 0.0408 | 0.0753 |
| Patient 27 | 0.0857 | 0.2452 | 0.0663 | 0.0357 | 0.1205 | 0.3192 | 0.1826 | 0.8431 |
| Patient 28 | 0.0779 | 0.0848 | 0.1578 | 0.0139 | 0.0155 | 0.0160 | 0.0820 | 0.2889 |
| Patient 29 | 0.0694 | 0.0446 | 0.0372 | 0.0112 | 0.0346 | 0.0684 | 0.0549 | 0.1072 |
| Patient 3 | 0.1408 | 0.3339 | 0.5278 | 0.0281 | 0.0506 | 0.5126 | 0.1049 | 0.0859 |
| Patient 30 | 0.2477 | 0.0862 | 0.0696 | 0.2720 | 0.2832 | 0.4631 | 0.3591 | 0.0320 |
| Patient 31 | 0.1001 | 0.0159 | 0.0746 | 0.2286 | 0.0081 | 0.0215 | 0.0108 | 0.1007 |
| Patient 32 | 0.0786 | 0.0614 | 0.0171 | 0.0134 | 0.1208 | 0.0196 | 0.0189 | 0.0272 |
| Patient 33 | 0.0204 | 0.1101 | 0.0719 | 0.0234 | 0.0815 | 0.0430 | 0.0240 | 0.0808 |
| Patient 34 | 0.1807 | 0.0715 | 0.3045 | 0.2866 | 0.5870 | 0.2122 | 0.0589 | 0.1932 |
| Patient 35 | 0.0087 | 0.0356 | 0.0201 | 0.1182 | 0.0188 | 0.0175 | 0.0835 | 0.0751 |
| Patient 36 | 0.0237 | 0.0469 | 0.0102 | 0.2018 | 0.0142 | 0.0025 | 0.0310 | 0.1450 |
| Patient 37 | 0.1728 | 0.2594 | 0.4353 | 0.1834 | 0.0587 | 0.0790 | 0.0953 | 0.0865 |
| Patient 38 | 0.1262 | 0.1103 | 0.2158 | 0.1837 | 0.1904 | 0.2029 | 0.1243 | 0.2056 |
| Patient 39 | 0.0567 | 0.1853 | 0.1089 | 0.3410 | 0.0692 | 0.0098 | 0.0300 | 0.1218 |
| Patient 4 | 0.3573 | 0.3937 | 0.1752 | 0.2357 | 0.1111 | 0.1578 | 0.4914 | 0.0200 |
| Patient 40 | 0.0272 | 0.1320 | 0.2923 | 0.0208 | 0.1699 | 0.0413 | 0.1891 | 0.0689 |
| Patient 41 | 0.1854 | 0.0677 | 0.1665 | 0.1194 | 0.0804 | 0.1323 | 0.1191 | 0.2125 |
| Patient 42 | 0.0618 | 0.0167 | 0.0506 | 0.2271 | 0.0145 | 0.0344 | 0.2094 | 0.0343 |
| Patient 43 | 0.0110 | 0.3243 | 0.0097 | 0.1998 | 0.2029 | 0.4222 | 0.1452 | 0.1194 |
| Patient 44 | 0.0805 | 0.1942 | 0.0834 | 0.1167 | 0.0415 | 0.0101 | 0.0515 | 0.0505 |
| Patient 45 | 0.0171 | 0.0446 | 0.1004 | 0.0662 | 0.0542 | 0.0401 | 0.1002 | 0.2604 |
| Patient 46 | 0.0054 | 0.0941 | 0.0687 | 0.3836 | 0.2691 | 0.1060 | 0.0340 | 0.1075 |
| Patient 47 | 0.0115 | 0.0045 | 0.0439 | 0.0235 | 0.0620 | 0.0056 | 0.0216 | 0.0335 |
| Patient 48 | 0.1953 | 0.0219 | 0.2111 | 0.3294 | 0.1606 | 0.0237 | 0.0626 | 0.0106 |
| Patient 49 | 0.0559 | 0.3038 | 0.1911 | 0.2152 | 0.0703 | 0.0096 | 0.1080 | 0.1059 |
| Patient 5 | 0.1893 | 0.1999 | 0.0150 | 0.4657 | 0.2570 | 0.0111 | 0.1788 | 0.1959 |
| Patient 50 | 0.1284 | 0.5918 | 0.1271 | 0.0527 | 0.3362 | 0.1220 | 0.0025 | 0.3798 |
| Patient 6 | 0.3506 | 0.2171 | 0.2401 | 0.2858 | 0.4298 | 0.4389 | 0.5856 | 0.5964 |
| Patient 7 | 0.0190 | 0.0915 | 0.0834 | 0.0895 | 0.0081 | 0.0368 | 0.1470 | 0.0378 |
| Patient 8 | 0.0239 | 0.0073 | 0.0736 | 0.1283 | 0.0032 | 0.0196 | 0.1387 | 0.1087 |
| Patient 9 | 0.0519 | 0.0519 | 0.0200 | 0.0888 | 0.1150 | 0.0192 | 0.0264 | 0.0448 |
| Control 72 | 0.0548 | 0.0701 | 0.0284 | 0.0776 | 0.0172 | 0.0286 | 0.0125 | 0.0447 |
| Control 73 | 0.0390 | 0.0860 | 0.0195 | 0.0147 | 0.1578 | 0.0199 | 0.3880 | 0.2834 |
| Control 74 | 0.1676 | 0.3630 | 0.4135 | 0.0051 | 0.3961 | 0.0532 | 0.4616 | 0.6589 |
| Control 75 | 0.0450 | 0.0681 | 0.1804 | 0.0143 | 0.0088 | 0.0511 | 0.2068 | 0.0213 |
| Control 76 | 0.1348 | 0.2254 | 0.0612 | 0.0017 | 0.0009 | 0.0973 | 0.1960 | 0.2415 |
| Patient 26 | 0.0107 | 0.0545 | 0.1613 | 0.2388 | 0.1420 | 0.0112 | 0.3439 | 0.2081 |

**Note**. CT SE = standard error of triplicate CT values. Missing values indicate single-replicate measurement.

## Supplementary Table S6. Intra-assay triplicate variability by gene

| Gene | N | Mean CV% | Median CV% | Min CV% | Max CV% |
| --- | --- | --- | --- | --- | --- |
| GAPDH | 98 | 1.215 | 0.765 | 0.006 | 5.778 |
| ADCY3 | 97 | 0.922 | 0.609 | 0.008 | 4.877 |
| CADM1 | 98 | 0.938 | 0.561 | 0.014 | 5.840 |
| DGKA | 96 | 1.248 | 0.770 | 0.014 | 6.668 |
| FAM46A | 98 | 1.027 | 0.472 | 0.004 | 5.575 |
| KIAA1539 | 97 | 0.862 | 0.450 | 0.009 | 4.476 |
| MARCKS | 97 | 1.410 | 0.922 | 0.021 | 7.342 |
| RAPH1 | 98 | 1.076 | 0.651 | 0.017 | 5.483 |

**Note**. CV% = (CT SD / CT Mean) × 100, where CT SD = CT SE × √3. N = number of samples with valid triplicate data. Mean CVs were below 2% for all targets, although individual sample-level maximum CVs exceeded 2% for some targets. N reflects all technically assayed samples (49 DD including Patient 10, 49 ND) prior to analytical exclusions. DD = depressive disorder.

## Supplementary Table S7. Robust bootstrap regression results for all genes.

| Gene | Model | β_Group | 95% CI Lower | 95% CI Upper | Bootstrap p |
| --- | --- | --- | --- | --- | --- |
| ADCY3 | Unadjusted | -0.064 | -0.617 | +0.543 | 0.7858 |
| ADCY3 | Age-adjusted | -0.260 | -0.794 | +0.229 | 0.3092 |
| CADM1 | Unadjusted | -0.384 | -1.045 | +0.294 | 0.2538 |
| CADM1 | Age-adjusted | -0.385 | -1.059 | +0.211 | 0.2016 |
| DGKA | Unadjusted | +0.132 | -0.423 | +0.762 | 0.6702 |
| DGKA | Age-adjusted | -0.123 | -0.622 | +0.359 | 0.6244 |
| FAM46A | Unadjusted | +0.203 | -0.358 | +0.786 | 0.4838 |
| FAM46A | Age-adjusted | -0.042 | -0.596 | +0.465 | 0.9042 |
| RAPH1 | Unadjusted | -0.222 | -0.808 | +0.406 | 0.4556 |
| RAPH1 | Age-adjusted | -0.304 | -0.880 | +0.216 | 0.2558 |
| KIAA1539 | Unadjusted | -0.054 | -0.642 | +0.555 | 0.8418 |
| KIAA1539 | Age-adjusted | -0.231 | -0.840 | +0.329 | 0.4478 |
| MARCKS | Unadjusted | +0.220 | -0.334 | +0.821 | 0.4516 |
| MARCKS | Age-adjusted | +0.174 | -0.371 | +0.691 | 0.5068 |

**Note**. Bootstrap regression (10,000 resamples) estimating group mean differences in ΔCT. β_Group = mean ΔCT difference (depressive disorder − non-depressed). Negative β indicates lower ΔCT (higher expression) in depressive disorders. Model 1: unadjusted. Model 2: age-adjusted. Computed using source-of-truth demographics.

## Supplementary Table S8. PCA of ΔCT values: variance and gene loadings

| PC | Var% | Cum% | ADCY3 | CADM1 | DGKA | FAM46A | RAPH1 | KIAA1539 | MARCKS | p (U) |
| --- | --- | --- | --- | --- | --- | --- | --- | --- | --- | --- |
| PC1 | 90.3 | 90.3 | 0.39 | 0.36 | 0.39 | 0.38 | 0.38 | 0.37 | 0.38 | 0.244 |
| PC2 | 3.5 | 93.9 | -0.01 | 0.75 | -0.09 | -0.23 | 0.33 | -0.42 | -0.31 | 0.006* |
| PC3 | 2.0 | 95.8 | 0.01 | 0.15 | 0.04 | -0.40 | -0.07 | 0.77 | -0.47 | 0.355 |

## Note. *p < 0.01. PC2 dominated by CADM1 (0.75) and RAPH1 (0.33). PC2 group effect attenuated after univariate age adjustment (ANCOVA p = 0.130; MANCOVA on all 7 genes, p = 0.156).

## Supplementary Table S9. Sex-stratified and nationality-stratified Mann-Whitney U results for all genes.

| Gene | Males p | Males d | Females p | Females d | Emiratis p | Emiratis d | Expats p | Expats d |
| --- | --- | --- | --- | --- | --- | --- | --- | --- |
| ADCY3 | 0.356 | -0.11 | 0.292 | +0.04 | 0.051 | -0.06 | 0.833 | -0.03 |
| CADM1 | 0.039* | -0.40 | 0.558 | -0.02 | 0.029* | -0.17 | 0.376 | -0.26 |
| DGKA | 0.607 | -0.00 | 0.907 | +0.22 | 0.222 | +0.04 | 0.643 | +0.12 |
| FAM46A | 0.793 | -0.05 | 0.145 | +0.37 | 0.950 | +0.23 | 0.316 | +0.07 |
| RAPH1 | 0.177 | -0.21 | 0.104 | -0.06 | 0.128 | +0.04 | 0.175 | -0.28 |
| KIAA1539 | 0.177 | +0.19 | 0.010* | -0.34 | 0.870 | +0.21 | 0.145 | -0.22 |
| MARCKS | 0.838 | -0.03 | 0.413 | +0.38 | 0.715 | +0.23 | 0.550 | +0.09 |

**Note**. All exploratory, uncorrected for multiple comparisons. d = Cohen's d (DD − ND; negative indicates lower ΔCT / higher expression in DD). *p < 0.05.

## Supplementary Table S10. Logistic regression classification of DD vs ND.

| Model | AUC ± SD | Accuracy | Sensitivity | Specificity | Interpretation |
| --- | --- | --- | --- | --- | --- |
| A: Genes only | 0.717 ± 0.016 | 67.6% | 62.5% | 73.5% | Modest discrimination |
| B: Genes + Age + Sex | 0.854 ± 0.007 | 78.6% | 75.0% | 83.7% | Age-driven improvement |
| C: Age + Sex only | 0.848 ± 0.003 | 76.9% | 75.0% | 79.6% | Genes add ΔAUC = 0.006 |

**Note**. 10-fold stratified cross-validation, averaged over 100 random seeds. All predictors standardised. Model C (age + sex only) serves as benchmark; ΔAUC indicates minimal added value of gene expression.

## Supplementary Table S11. Logistic regression standardised coefficients (genes-only model).

| Gene | Standardised β | Interpretation |
| --- | --- | --- |
| ADCY3 | -0.533 | Lower ΔCT → DD |
| CADM1 | -0.939 | Lower ΔCT → DD |
| DGKA | +1.116 | Higher ΔCT → DD |
| FAM46A | +1.161 | Higher ΔCT → DD |
| RAPH1 | -1.271 | Lower ΔCT → DD |
| KIAA1539 | -0.355 | Lower ΔCT → DD |
| MARCKS | +0.734 | Higher ΔCT → DD |

**Note**. Standardised β coefficients from the genes-only logistic regression model. Positive β indicates higher ΔCT (lower expression) associated with DD classification. DD = depressive disorder; ND = non-depressed.

## Supplementary Table S12. Spearman correlations between gene expression (ΔCT) and depression severity.

| Gene | BDI-II ρ | BDI-II p | BDI-II p_adj | PHQ-9 ρ | PHQ-9 p | PHQ-9 p_adj |
| --- | --- | --- | --- | --- | --- | --- |
| ADCY3 | -0.229 | 0.024* | 0.083 | -0.188 | 0.066 | 0.153 |
| CADM1 | -0.280 | 0.005** | 0.076 | -0.243 | 0.017* | 0.077 |
| DGKA | -0.084 | 0.412 | 0.671 | -0.050 | 0.625 | 0.763 |
| FAM46A | +0.022 | 0.833 | 0.833 | +0.023 | 0.823 | 0.833 |
| RAPH1 | -0.246 | 0.015* | 0.077 | -0.198 | 0.052 | 0.145 |
| KIAA1539 | -0.081 | 0.431 | 0.671 | -0.060 | 0.562 | 0.763 |
| MARCKS | +0.046 | 0.654 | 0.763 | +0.097 | 0.343 | 0.671 |

**Note**. Full sample N = 97. ρ = Spearman rank correlation. Negative ρ indicates higher expression (lower ΔCT) associated with higher depression scores. p_adj = Benjamini-Hochberg corrected across 14 tests. *p < 0.05 uncorrected. **p < 0.01 uncorrected.

## Supplementary Table S13. Within-DD gene expression–depression severity correlations.

| Gene | BDI-II ρ | BDI-II p | PHQ-9 ρ | PHQ-9 p |
| --- | --- | --- | --- | --- |
| ADCY3 | -0.147 | 0.3193 | -0.112 | 0.4489 |
| CADM1 | -0.248 | 0.0897 | -0.132 | 0.3728 |
| DGKA | -0.003 | 0.9848 | +0.035 | 0.8141 |
| FAM46A | -0.247 | 0.0910 | -0.234 | 0.1089 |
| RAPH1 | -0.174 | 0.2359 | -0.063 | 0.6705 |
| KIAA1539 | +0.009 | 0.9538 | +0.150 | 0.3104 |
| MARCKS | -0.056 | 0.7061 | +0.081 | 0.5865 |

**Note**. Spearman rank correlations within the DD group only (N = 48, excluding Patient 10). ρ = Spearman correlation coefficient. DD = depressive disorder.

**Supplementary Table S14.** Severity-stratified Mann-Whitney U comparisons of ΔCT between depressive-disorder and non-depressed control groups for all seven candidate genes.

| Gene | Case stratum | n (case) | n (ND) | U | Mann-Whitney p | Cohen's d | Cliff's δ |
| --- | --- | --- | --- | --- | --- | --- | --- |
| *ADCY3* | Full depressive disorder | 48 | 49 | 977 | 0.152 | −0.044 | −0.169 |
|  | BDI-II ≥20 | 40 | 49 | 756 | 0.065 | −0.111 | −0.229 |
|  | BDI-II ≥29 | 31 | 49 | 570 | 0.062 | −0.146 | −0.250 |
| *CADM1* | Full depressive disorder | 48 | 49 | 903 | 0.049 | −0.225 | −0.232 |
|  | BDI-II ≥20 | 40 | 49 | 714 | 0.029 | −0.281 | −0.271 |
|  | BDI-II ≥29 | 31 | 49 | 511 | 0.014 | −0.338 | −0.327 |
| *DGKA* | Full depressive disorder | 48 | 49 | 1116 | 0.668 | 0.088 | −0.051 |
|  | BDI-II ≥20 | 40 | 49 | 916 | 0.600 | 0.048 | −0.065 |
|  | BDI-II ≥29 | 31 | 49 | 692 | 0.508 | 0.002 | −0.089 |
| *FAM46A* | Full depressive disorder | 48 | 49 | 1284 | 0.438 | 0.137 | 0.092 |
|  | BDI-II ≥20 | 40 | 49 | 943 | 0.763 | 0.040 | −0.038 |
|  | BDI-II ≥29 | 31 | 49 | 712 | 0.643 | 0.020 | −0.063 |
| *KIAA1539* | Full depressive disorder | 48 | 49 | 1046 | 0.350 | −0.035 | −0.111 |
|  | BDI-II ≥20 | 40 | 49 | 861 | 0.328 | −0.066 | −0.121 |
|  | BDI-II ≥29 | 31 | 49 | 682 | 0.447 | −0.096 | −0.102 |
| *MARCKS* | Full depressive disorder | 48 | 49 | 1224 | 0.732 | 0.151 | 0.041 |
|  | BDI-II ≥20 | 40 | 49 | 1002 | 0.859 | 0.111 | 0.022 |
|  | BDI-II ≥29 | 31 | 49 | 750 | 0.929 | 0.053 | −0.013 |
| *RAPH1* | Full depressive disorder | 48 | 49 | 905 | 0.051 | −0.143 | −0.230 |
|  | BDI-II ≥20 | 40 | 49 | 709 | 0.026 | −0.199 | −0.277 |
|  | BDI-II ≥29 | 31 | 49 | 532 | 0.025 | −0.250 | −0.300 |

**Note.** All analyses are based on the gene-expression analytic sample after exclusion of one depressive-disorder participant (Patient 10) on the basis of technical sample failure (see Methods). The non-depressed control group (n = 49) is held constant across all comparisons; only the depressive-disorder group is restricted by severity stratum. Strata: Full depressive disorder = all 48 analytic-sample participants (mean BDI-II 32.5); BDI-II ≥ 20 = moderate-to-severe stratum (n = 40; mean BDI-II 35.8); BDI-II ≥ 29 = severe stratum (n = 31; mean BDI-II 38.6). U = Mann-Whitney U statistic; d = Cohen's d (depressive-disorder minus non-depressed; negative d indicates lower ΔCT, i.e. higher expression in the depressive-disorder group); δ = Cliff's delta. All p-values are uncorrected for multiple comparisons; the severity-stratified analyses are exploratory sensitivity analyses pre-specified to address potential dilution of a depression-specific signal by diagnostically milder sub-strata. CADM1 strengthened monotonically across severity strata (full sample p = 0.049, d = −0.225; moderate-severe p = 0.029, d = −0.281; severe p = 0.014, d = −0.338), as did RAPH1 (p = 0.051, 0.026, 0.025) and ADCY3 (p = 0.152, 0.065, 0.062). No comparison would survive Benjamini-Hochberg correction across the 21 tests reported in this table.
